# PRMT5/WDR77 Enhances the Proliferation of Squamous Cell Carcinoma via the ΔNp63α-p21 Axis

**DOI:** 10.3390/cancers16223789

**Published:** 2024-11-11

**Authors:** Heng Liang, Matthew L. Fisher, Caizhi Wu, Carlos Ballon, Xueqin Sun, Alea A. Mills

**Affiliations:** 1 Cold Spring Harbor Laboratory, Cold Spring Harbor, NY 11724, USA; hliang@cshl.edu (H.L.); fisher@cshl.edu (M.L.F.); wuc@cshl.edu (C.W.); ballon@cshl.edu (C.B.); xsun@sbpdiscovery.org (X.S.); 2 Molecular and Cell Biology Graduate Program, Stony Brook University, Stony Brook, NY 11794, USA

**Keywords:** PRMT5, WDR77, squamous cell carcinoma, cell proliferation, ΔNp63α, p21

## Abstract

Protein arginine methyltransferase 5 (PRMT5) is a critical oncogenic factor in various cancers, and its inhibition has shown promise in suppressing tumor growth. However, the role of PRMT5 in squamous cell carcinoma (SCC) remains largely unexplored. In this study, we analyzed SCC patient data from The Cancer Genome Atlas (TCGA) and the Cancer Dependency Map (DepMap) to investigate the relationship between PRMT5 and SCC proliferation. We employed competition-based cell proliferation assays, 3-(4,5-dimethylthiazol-2-yl)-2,5-diphenyl-2H-tetrazolium bromide (MTT) assays, flow cytometry, and in vivo mouse modeling to examine the regulatory roles of PRMT5 and its binding partner WDR77 (WD repeat domain 77). We identified downstream targets, including the p63 isoform ΔNp63α and the cyclin-dependent kinase inhibitor p21, through single-cell RNA-seq, RT-qPCR, and Western blot analyses. Our findings demonstrate that upregulation of PRMT5 and WDR77 correlates with the poor survival of head and neck squamous cell carcinoma (HNSCC) patients. PRMT5/WDR77 regulates the HNSCC-specific transcriptome and facilitates SCC proliferation by promoting cell cycle progression. The PRMT5 and WDR77 stabilize the ΔNp63α Protein, which in turn, inhibits p21. Moreover, depletion of PRMT5 and WDR77 repress SCC in vivo. This study reveals for the first time that PRMT5 and WDR77 synergize to promote SCC proliferation via the ΔNp63α-p21 axis, highlighting a novel therapeutic target for SCC.

## Simple Summary

Protein arginine methyltransferase 5 (PRMT5) is known to be oncogenic in many cancers, including squamous cell carcinoma (SCC). Our analyses of multiple public databases revealed that PRMT5 overexpression correlates with poor survival in SCC patients and is essential to the survival of SCC cell lines. This study focused on understanding how PRMT5 and its binding partner, WDR77 (WD repeat domain 77), regulate SCC cell growth, particularly through the p63 ΔNp63α isoform, a key factor in SCC. Furthermore, PRMT5 depletion inhibited SCC proliferation by inducing cell cycle arrest in the G1 phase. Additionally, we showed that PRMT5 and WDR77 stabilized ΔNp63α protein expression, which in turn inhibited p21 (cyclin-dependent kinase inhibitor 1). These findings provide new insights into the potential of targeting PRMT5 as a therapeutic strategy for SCC.

## 1. Introduction

Squamous cell carcinoma (SCC) is among the most prevalent cancers, affecting various regions such as the skin, lungs, head, neck, and cervix. SCCs are classified based on their primary tumor sites, with head and neck squamous cell carcinoma (HNSCC) being the sixth most common cancer globally [1]. Major risk factors for HNSCC include exposure to alcohol, tobacco, and human papillomavirus (HPV). Typically, HPV-negative cases tend to have worse survival outcomes than HPV-positive cases [2,3]. Moreover, one common feature shared by different SCC classifications, including HNSCC and cutaneous squamous cell carcinoma (CSCC), is the over-proliferation of epithelial basal cells that maintain the squamous stratified epithelium. These two subtypes, HNSCC and CSCC, more frequently originate from stratified squamous epithelial structures compared to other SCC subtypes [4].

Numerous studies have highlighted the significant role of protein methyltransferases in the genetic alterations observed in cancers [5,6]. There are two main types of protein methyltransferases, namely protein arginine methyltransferase (PRMTs) and protein lysine methyltransferase (PKMTs) [5]. Among them, PRMT5 has been identified as an oncogenic factor in various cancers [6]. PRMT5 has two key domains: an N-terminal domain with a TIM-barrel structure for protein interactions and a SAM-dependent MTase PRMT-type domain responsible for the methylation reaction [7]. The WD-repeat protein WDR77, also known as MEP50, serves as the primary binding partner of PRMT5. Together, they form the PRMT5/WDR77 complex, which exhibits robust methyltransferase activity and stability [7,8].

The tumor protein p63, a member of the p53 family of transcription factors, regulates key cellular functions, including proliferation [9] and senescence [10]. Among the many p63 isoforms, ΔNp63α is predominantly expressed in the basal layer of squamous stratified epithelia [11]. ΔNp63α exerts a pro-proliferative effect through transcriptional repression of the cyclin-dependent kinase (CDK) inhibitor p21 [12,13]. While the significance of ΔNp63α in SCC has been well established, mechanisms that enhance ΔNp63α expression and maintain proliferation in SCC remain largely unknown.

In this study, we investigated the epigenetic mechanisms driving cellular proliferation in epithelial basal cells, focusing on PRMT5/WDR77-mediated SCC proliferation. We identified a novel PRMT5/WDR77-ΔNp63α-p21 signaling axis that plays a crucial role in sustaining SCC proliferation.

## 2. Materials and Methods

### 2.1. Cell Culture

Human FaDu and Cal-33 cell lines (HNSCC cell lines) were generously provided by Leif Ellisen, while the human HSC-5 line (a CSCC cell line from an ulcerated tumor on the upper part of the right ear [14]) was obtained from Sekisui Xenotech, LLC (Kansas City, KS, USA). All cell lines were maintained in Dulbecco’s Modified Eagle’s Medium (DMEM, Corning, Corning, NY, USA, Cat. No. 17-207-CV) supplemented with 10% fetal bovine serum (FBS, Corning, Corning, NY, USA, Cat. No. 35-010-CV) and 1% penicillin–streptomycin. Trypsin was purchased from Corning (Cat. No. 25-054-CI, Corning, NY, USA). All cell lines were authenticated and tested negative for mycoplasma by the University of Arizona Genetics Core on 2 April 2024.

### 2.2. Tumor Microarrays

Tissue arrays of SCC were obtained from US Biomax (Derwood, MD, USA, Cat. No. HN804). Expression of PRMT5 and p63 was assessed as previously described [15]. The PRMT5 antibody (Cell Signaling, Danvers, MA, USA, Cat. No. 79998) was used at a 1:1000 concentration, and the ΔNp63α antibody (Cell Signaling, Danvers, MA, USA, Cat. No. 13109) was used at a 1:500 concentration.

### 2.3. Statistical Analysis Using TCGA, DepMap, and Domain-Focused CRISPR Screening Data

Analyses of TCGA and DepMap databases involved downloading the data followed by manipulation and visualization using R (Version 4.4.0, 2024-04-24) [16]. Detailed statistical analyses are described in the Figure legends. All data are open-access and do not require authorization, ensuring the protection of patient privacy.

The Domain-focused CRISPR screening data were obtained from several studies [17,18,19], and R was used for data manipulation and visualization.

### 2.4. RNA-seq and Single-Cell RNA-seq Data Analysis

RNA-sequencing (RNA-seq) analyses were conducted on three biological replicates from HSC-5 cells with complete PRMT5KO or WDR77KO that had been validated using qRT-PCR. Total RNA was extracted, purified, and barcoded using the TruSeq RNA Library Prep Kit v2 (Illumina Inc., San Diego, CA, USA). Sequencing was performed at the Cold Spring Harbor Laboratory (CSHL) Next Generation Sequencing Shared Resource using 76nt single-end mode. Subsequently, data analysis was executed via a Linux-based server at CSHL. Mapping was accomplished using STAR (Spliced Transcript Alignment to a Reference Software, hg38, Version 2.7.11b) [20], and counts were generated using htseq-count (Version 2.0.3) [21]. Differential analysis was performed using DESeq2 (Version 3.20) [22] in R. Gene Set Enrichment Analysis (GSEA) [23] was conducted using fgsea (Version 3.20) [24], and before running the package fgsea, all genes were ranked by Log2Fold change. Data manipulation and visualization were carried out using R packages such as dplyr (Version 1.1.4) [25] and ggplot2 (Version 3.5.1) [26].

Single-cell RNA sequencing (scRNA-seq) data were processed using the Seurat v5 pipeline [27]. Data manipulation and visualization were performed using R packages, including dplyr and ggplot2.

### 2.5. Plasmid Construction, Virus Transduction, and Infections

Plasmid construction, virus transduction, and infection were performed using previously described protocols [17]. The sequences of the sgRNAs were provided in Supplementary Table S1.

The wild-type *PRMT5* (Catalog ID: MHS6278-202829982) and *WDR77* (Catalog ID: MHS6278-202756033) cDNAs were obtained from Horizon Discovery (Cambridge, UK). Details of mutations for *PRMT5* cDNA (PRMT5 CR) and *WDR77* cDNA (WDR77 CR) were provided in Supplementary Figure S4B. CRISPR-resistant mutations were introduced based on the codon frequency of the human genome [28].

### 2.6. Competition-Based Cell-Proliferation Assays

Individual small guide RNAs targeting *PRMT5* and *WDR77* were cloned into GFP expression vectors as previously described [17]. Constructs were packaged into lentivirus and used to infect Cas9-expressing cell lines, including FaDu, HSC5, and Cal33. Guide RNA expression and gene depletion were monitored by measuring GFP expression over 24 days (8 passages). Guide RNAs targeting *ROSA26* and *CDK1* were used as negative and positive controls, respectively. The depletion assays were run in triplicate. GFP levels were measured using the Guava Easycyte HT instrument (Millipore, Burlington, MA, USA) 48 h post-infection, with the initial GFP percentages having been adjusted to 30–70%.

### 2.7. MTT Assays

The cells were first seeded into 96-well plates at a concentration of 5000 cells per well. One day after plating, cells were treated with either DMSO (vehicle) (Sigma-Aldrich, St. Louis, MO, USA, Cat. No. D4540-1L) or 4 μmol/L PF-06939999 (Chemietek, Indianapolis, IN, Cat. No. CT-PF0693). The media were changed every 24 h, and a fresh vehicle or inhibitor was added. After 48 h of treatment, cells were grown in fresh medium with MTT solution for 4 h at 37 °C, followed by solubilization in 50 µL DMSO. The absorbance of the final purple formazan solution was assessed using a GloMax^®^ Discover Microplate Reader (GM3000, Promega, Madison, WI, USA) at a wavelength of 540 nm.

For CRISPR-mediated depletion and rescue experiments, the cells were infected with a virus encoding the sgRNAs, including sgNeg, sgPRMT5-1, or sgWDR77-2. After selection with G418 (Sigma-Aldrich, St. Louis, MO, USA, CAS Number 108321-42-2), the cells were seeded at a concentration of 5000 cells per well in 96-well plates, and after 48 h, cell concentrations were evaluated using MTT assays as described above. Triplicate wells were prepared for each condition.

### 2.8. Flow Cytometry

Cells were fixed using Click-iT^®^ fixative buffer (4% paraformaldehyde in PBS) and permeabilized using Click-iT^®^ saponin-based permeabilization and wash reagent from the Click-iT™ Plus EdU Flow Cytometry Assay Kits (Invitrogen, Waltham, MA, USA, Cat. No. C10634). Samples were then stored at 4 °C until processing. Prior to staining with FxCycle™ Violet Stain (Invitrogen, Waltham, MA, USA, Cat. No. F10347), the cells were counted and adjusted to a concentration of 1 × 10^6^ cells/mL in PBS containing 1% BSA. After incubation for 30 min at room temperature, protected from light, the samples were subjected to flow cytometry using the violet 405 nm excitation on a BD LSR Dual Fortessa Cell Analyzer (BD Biosciences, San Jose, CA, USA). FlowJo software (Becton Dickinson, Version 10.9) was used for data analysis.

### 2.9. Western Blotting

Protein lysates were prepared using RIPA lysis buffer, followed by centrifugation for 15 min to collect the supernatants. Proteins of equal amounts were loaded onto denaturing and reducing 10% polyacrylamide gels for electrophoresis and subsequently transferred to nitrocellulose membranes. Membranes were blocked by 5% non-fat milk for 1 h at room temperature and incubated overnight at 4 °C with the appropriate primary antibody. The list of antibodies used in this study includes: PRMT5 (Cell Signaling, Danvers, MA, USA, Cat. No. 79998), WDR77 (Cell Signaling, Danvers, MA, USA, Cat. No. 2823), ΔNp63α (Cell Signaling, Danvers, MA, USA, Cat. No. 39692), p21 (Santa Cruz Biotechnology, Dallas, TX, USA, Cat. No. sc-71811), β-Actin (Santa Cruz Biotechnology, Dallas, TX, USA, Cat. No. sc-47778), and HSC70 (Santa Cruz Biotechnology, Dallas, TX, USA, Cat. No. sc-7298).

After being washed with TBST three times, the membranes were incubated with the corresponding secondary antibodies (diluted 1:2500) for 1 h at room temperature. Secondary antibody binding was visualized using chemiluminescence detection technology with the SuperSignal West Dura Extended Duration substrate purchased from Thermo Fisher Scientific (Waltham, MA, USA, Cat. No. 34076). Western blot images were obtained via the Odyssey Fc Imaging System from LICORbio (Lincoln, NE, USA). Full images were provided in Supplementary Figure S7.

### 2.10. Quantitative Real-Time PCR

Total RNA was extracted using the RNeasy Plus Mini Kit (Qiagen, Hilden, Germany, Cat. No. 74134). Reverse transcription of 2.5 μg of total RNA was then performed using the Superscript III First-Strand Synthesis System (Invitrogen, Waltham, MA, USA, Cat. No. 18080-51) to synthesize cDNA. Samples and corresponding primers were processed using the Power SYBR™ GREEN PCR Master Mix (Thermo Fisher, Waltham, MA, USA, Cat. No. 4367659), and Ct values for each sample were obtained with the QuantStudio™ 6 Flex Real-Time PCR System (Thermo Fisher, Waltham, MA, USA). Expression signals were normalized to GAPDH mRNA levels to determine relative expression levels (2^−∆∆Ct^ method). The sequences of the PCR primers were provided in Supplementary Table S1.

### 2.11. Tumor Xenografts

Animal experiments were approved by the Institutional Animal Care and Use Committee (IACUC).

FaDu cells (5 × 10⁴) were suspended in 100 μL of DMEM media and mixed with Matrigel in a 1:1 ratio (Corning, Corning, NY, USA, Cat. No. 354234). An equal volume of mixture was injected subcutaneously into both rear flanks of nude mice (Jackson, NU/J, Bar Harbor, ME, USA, Cat. No. 002019, homozygous for *Foxn1nu*) using 23-gauge needles attached to 1 cc syringes (n = 4/each group). Cells and syringes were kept on ice throughout the procedure to prevent the Matrigel from solidifying.

Once tumors were observed, their growth was closely monitored daily. The tumors were weighed and collected after 22 days when the diameters of some tumors exceeded 20 mm as measured by calipers. To ensure blinding, the mice were initially labeled as groups 1, 2, and 3, without knowledge of the treatment groups until all measurements were completed. Further details of the protocol are available in our previous lab publication [15]. Raw images of the tumors were provided in Supplementary Figure S8.

### 2.12. Statistical Analysis

All quantitative data are presented as the means ± S.D. of three biologically independent experiments. Statistical analyses were performed using GraphPad Prism 10 (GraphPad, Boston, MA, USA, Version 10.4.0). Significance was determined as indicated in the Figure legends. *p* < 0.05 was considered significant.

## 3. Results

### 3.1. PRMT5 and WDR77 Upregulation Correlates with Poor Survival in HNSCC Patients

**Figure 1:**
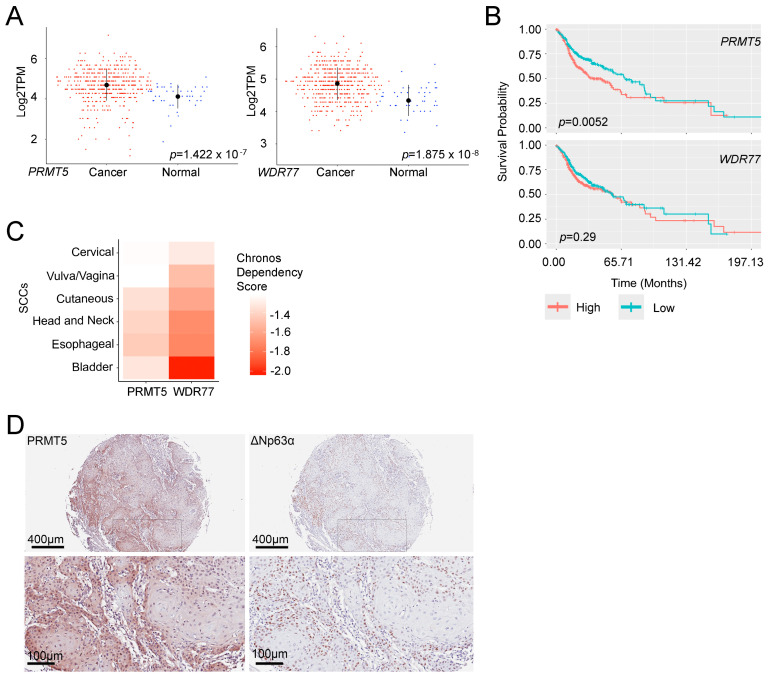
*PRMT5* is inversely correlated with SCC survival. (**A**) Statistical analysis of the TCGA-HNSC database comparing the expression of *PRMT5* (left) and *WDR77* (right) in cancer (red) vs. normal (blue) groups, utilizing Wilcoxon Rank-Sum Tests. Patient counts: HNSCC (513 tumors and 44 normal). Black dots represent median expression levels. (**B**) Kaplan–Meier survival curve for *PRMT5* and *WDR77* expression in the TCGA-HNSC database (n = 513). Patients were categorized by median *PRMT5* (upper panel) and *WDR77* (lower panel) expression levels. Statistical significance was determined using the Log-Rank Tests, with visualization provided by the ggsurvplot function from the survminer R package [29]. (**C**) Statistical analysis of DepMap data and display of Chronos Dependency Scores for six subtypes of SCC. Chronos Dependency Scores, derived from cell depletion assays, indicate gene essentiality, where lower (more negative) scores reflect higher gene essentiality. (**D**) Immunohistochemical staining for PRMT5 (left) and ΔNp63α (right) in HNSCC patient specimens. The top panels show low-magnification views. Scale bars: 400 µm (top) and 100 µm (bottom).

To determine the expression levels of *PRMT5* and its binding partner *WDR77* in HNSCC patients, we analyzed data from 557 cases (513 tumors and 44 normal samples) from the TCGA-HNSC project. Our analysis revealed significant upregulation of both *PRMT5* and *WDR77* mRNA levels in tumor samples compared to normal samples from patients with HNSCC (Figure 1A). Furthermore, we observed positive correlations between the mRNA levels of *PRMT5*, *WDR77*, and *TP63*, with a notably stronger correlation between *PRMT5* and *TP63* (R = 0.45) than between *PRMT5* and *WDR77* (R = 0.3) (Supplementary Figure S1A). Higher *PRMT5* expression was significantly associated with poor survival in HNSCC patients, whereas high *WDR77* was not associated with survival (Figure 1B). Additionally, DepMap data supported the essential role of both PRMT5 and WDR77 for the survival of SCC cells, with a strong correlation in their essentiality across different types of SCC (Figure 1C, Supplementary Figure S1B). Domain-focused CRISPR screening in cancer lines including HSC5 revealed that control cells outnumbered PRMT5KO cells, indicating that PRMT5 was essential for viability (Supplementary Figure S1C). We also examined the protein levels of PRMT5 and ΔNp63α in HNSCC patient samples, which revealed that PRMT5 and ΔNp63α were co-expressed within the proliferative basal layer of the stratified squamous epithelium of the tumors; this cell population consisted of less-differentiated basal-like cells (Figure 1D). These findings indicate that high-level expression of *PRMT5*, *WDR77*, and *ΔNp63α* occur in HNSCC and that both PRMT5 and WDR77 are essential for HNSCC cell survival.

### 3.2. PRMT5 and WDR77 Regulate the HNSCC-Specific Transcriptome

We employed RNA-seq to define the transcriptomes of *PRMT5* and *WDR77* CRISPR-depleted cells. We confirmed the reduced expression levels of *PRMT5* and *WDR77* in the RNA-seq data (Supplementary Figure S2A). We validated that each sample displayed distinct features between different conditions in the PCA plots (Supplementary Figure S2B) prior to DESeq2 analysis. This analysis identified genes that were significantly deregulated in *PRMT5* and *WDR77* knockout cells (KO) (Supplementary Figure S2C). We found that 1405 genes were significantly upregulated in PRMT5KO and 214 were significantly upregulated in WDR77KO, whereas 1092 genes were significantly downregulated in PRMT5KO and 425 genes were significantly downregulated in WDR77KO.

**Figure 2:**
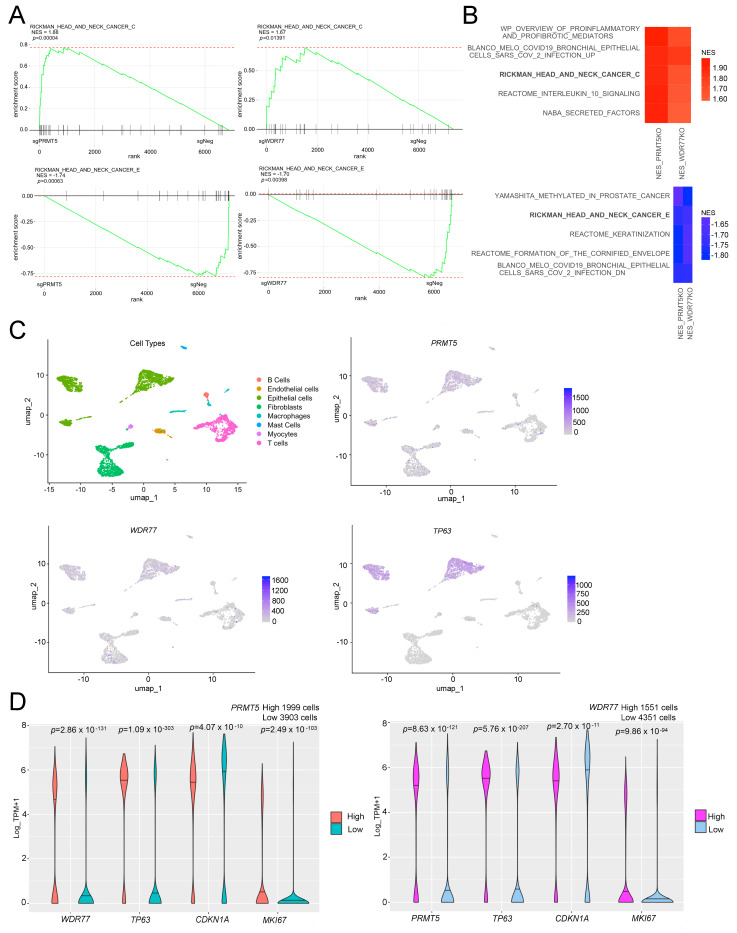
*PRMT5* and *WDR77* regulate the HNSCC-specific transcriptome. (**A**) GSEA plots identified genes affected in both PRMT5KO and WDR77KO, with notably enriched RICKMAN_HEAD_AND_NECK_CANCER C and E gene sets. (**B**) Heatmaps of GSEA results for both PRMT5KO and WDR77KO, highlighting the top five positively and negatively enriched gene sets. (**C**) UMAP visualizations depict cell types for each cluster and expression patterns of *PRMT5*, *WDR77,* and *TP63*. (**D**) Comparison of *PRMT5* (left) or *WDR77* (right), along with *TP63*, *CDKN1A,* and *MKI67* gene expression, in *PRMT5^High^* vs. *PRMT5^Low^* (left) and in *WDR77^High^* vs. *WDR77^Low^* (right) subgroups in single HNSCC cells. *p*-values were calculated using the Wilcoxon Rank-Sum Tests, and the median (50th percentile) for each dataset is denoted by a solid line.

To identify genes co-regulated by both PRMT5 and WDR77, we generated Venn diagrams to compare the overlap between PRMT5KO and WDR77KO (Supplementary Figure S2D, upper panel). We found that 104 genes were significantly upregulated, and 249 genes were significantly downregulated in both PRMT5KO and WDR77KO, which represented 7.4% and 22.8%, respectively, of the genes deregulated by PRMT5 depletion, or 48.6% and 58.6%, respectively, of the genes deregulated by WDR77 depletion. Contingency tables revealed a significant association between the two KOs for both upregulated and downregulated genes (Fisher’s Exact Test, adjusted *p* < 0.05) (Supplementary Figure S2D, lower panel). Consequently, we focused on these PRMT5/WDR77-deregulated genes and conducted GSEA using the C2 gene sets (n = 7233) from the Human Molecular Signatures Database (MSigDB) [23,30,31]. Interestingly, two RICKMAN_HEAD_AND_NECK_CANCER gene signatures [32] ranked as the top gene sets (Figure 2A). The RICKMAN_HEAD_AND_NECK_CANCER dataset was categorized into groups A–F that represented distinct gene signatures. The previously described gene signature groups of the RICKMAN_HEAD_AND_NECK_CANCER dataset correspond to gene ontologies such as cell motility and cell differentiation (group A); ECM and tissue development (group B); tissue development and adhesion proteins (group C); immune response (group D); cell differentiation (group E); and muscle contraction development (group F), and each of these gene sets had been defined as hallmark genes deregulated in HNSCC. Among these gene signatures, we found that groups C (associated with tissue development and adhesion proteins) and E (associated with cell differentiation) were the top-ranked gene sets enriched in PRMT5KO and WDR77KO, respectively (Figure 2B), supporting the potential roles of *PRMT5* and *WDR77* in HNSCC-specific pathways. Additionally, we found 49 gene sets positively enriched, and 96 gene sets negatively enriched in both PRMT5KO and WDR77KO groups, all with an adjusted *p* < 0.05 (Supplementary Figure S2E). These findings indicate that genes co-regulated by *PRMT5* and *WDR77* are deregulated in HNSCC.

We further analyzed single-cell RNA-seq (scRNA-seq) data from primary tumors of 18 treatment-naïve, HPV-negative HNSCC patients, from a study performed at the Broad Institute of Harvard and MIT [33]. This dataset, which included 5902 cells, was classified into 18 clusters based on their gene expression profiles (Supplementary Figure S3A). After annotating the clusters by the expression of known marker genes, we identified B cells, endothelial cells, epithelial cells, fibroblasts, macrophages, mast cells, myocytes, and T cells (Supplementary Figure S3B). *PRMT5*, *WDR77*, and *TP63* were predominantly expressed in epithelial cells (Figure 2C, Supplementary Figure S3C). Based on *PRMT5* expression levels, we divided the cells into *PRMT5^High^* (n = 1999) and *PRMT5^Low^* (n = 3903) groups (Figure 2D). *WDR77* (*p* = 2.86 × 10^−131^), *TP63* (*p* = 1.09 × 10^−303^), and *MKI67* (*p* = 2.49 × 10^−103^) were significantly enriched in *PRMT5^High^* HNSCC cells, whereas *CDKN1A* (*p* = 4.07 × 10^−10^) was significantly enriched in *PRMT5^Low^* HNSCC cells (Figure 2D, left panel). A similar pattern was observed when we sub-grouped cells based on *WDR77* levels (*WDR77^High^*, n = 1551 and *WDR77^Low^*, n = 4351) (Figure 2D, right panel). The *p*-values for this comparison were *PRMT5* (*p* = 8.63 × 10^−121^), *TP63* (*p* = 5.76 × 10^−207^), *MKI67* (*p* = 9.86 × 10^−94^), and *CDKN1A* (*p* = 2.70 × 10^−11^). These findings highlight a strong association between *PRMT5*, *WDR77*, and *TP63* (a marker characteristic of HNSCC), within single cells of HNSCC. Thus, the match to hallmark gene signatures and the association with p63 indicate that PRMT5 and WDR77 work together to co-regulate an HNSCC-specific transcriptome.

### 3.3. PRMT5 and WDR77 Mediate SCC Proliferation by Promoting Cell Cycle Progression

**Figure 3:**
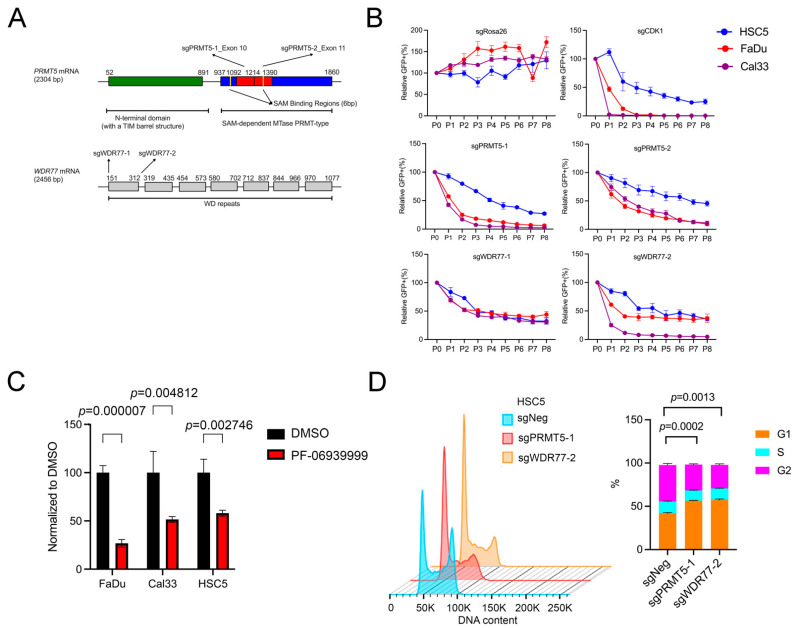
Depletion of PRMT5 or WDR77 inhibits cell proliferation. (**A**) CRISPR-mediated depletion of *PRMT5* or *WDR77* in SCC cells using four sgRNAs (two for each gene). (**B**) Cellular competition-based GFP dropout assays in HSC5, FaDu, and Cal33 cells treated with sgRosa26, sgCDK1, sgPRMT5-1, sgPRMT5-2, sgWDR77-1, and sgWDR77-2. Normalized to P0. (**C**) MTT-based proliferation assays in the indicated SCC cell lines. Cells were treated with DMSO or the PRMT5 inhibitor PF-06939999 (4 μmol/L) for 48 h. Data were normalized to DMSO controls (n  =  3 biologically independent replicates). The *p*-values were calculated using two-tailed unpaired Student’s *t*-tests. (**D**) Flow cytometry of HSC5 cells treated with sgNeg, sgPRMT5-1, and sgWDR77-2 (n  =  3 biologically independent samples). The *p*-values were calculated using Two-Way ANOVA.

To assess the functional role of PRMT5 and WDR77 in SCC, we designed sgRNAs targeting exons encoding the catalytic domain of *PRMT5*, and the first WD repeat of *WDR77* (Figure 3A). CRISPR-mediated depletion indicated that loss of either PRMT5 or WDR77 reduced cell survival across SCC cell lines (Figure 3B). The on-target efficacies of the sgRNAs were confirmed through cDNA rescue experiments (Supplementary Figure S4A,B), and the protein levels in KOs and rescue cells were verified (Supplementary Figure S4C). Overexpression of PRMT5 in WDR77KO cells failed to rescue WDR77 protein expression (Supplementary Figure S4C) or cell survival (see Supplementary Figure S4A); the same was also true in that overexpression of WDR77 failed to rescue the effect of PRMT5 depletion, indicating that both PRMT5 and WDR77 were essential for cell survival. To further validate PRMT5 as a potential therapeutic target, we treated the cells with the PRMT5 inhibitor PF-06939999 [34], which similarly resulted in impaired cell survival (Figure 3C). To elucidate how the loss of PRMT5 or WDR77 affected cell proliferation, we analyzed cell cycle progression in both PRMT5- and WDR77-depleted cells and found that the absence of PRMT5 or WDR77 significantly arrested cells at the G1 phase of the cell cycle (Figure 3D, Supplementary Figure S4D). Collectively, our findings demonstrate that PRMT5 and WDR77 are both needed to promote SCC proliferation and that depletion of either PRMT5 or WDR77 inhibits SCC proliferation by inducing cell cycle arrest in the G1 phase.

### 3.4. PRMT5 and WDR77 Stabilize the ΔNp63α Protein, Which, in Turn, Inhibits p21

**Figure 4:**
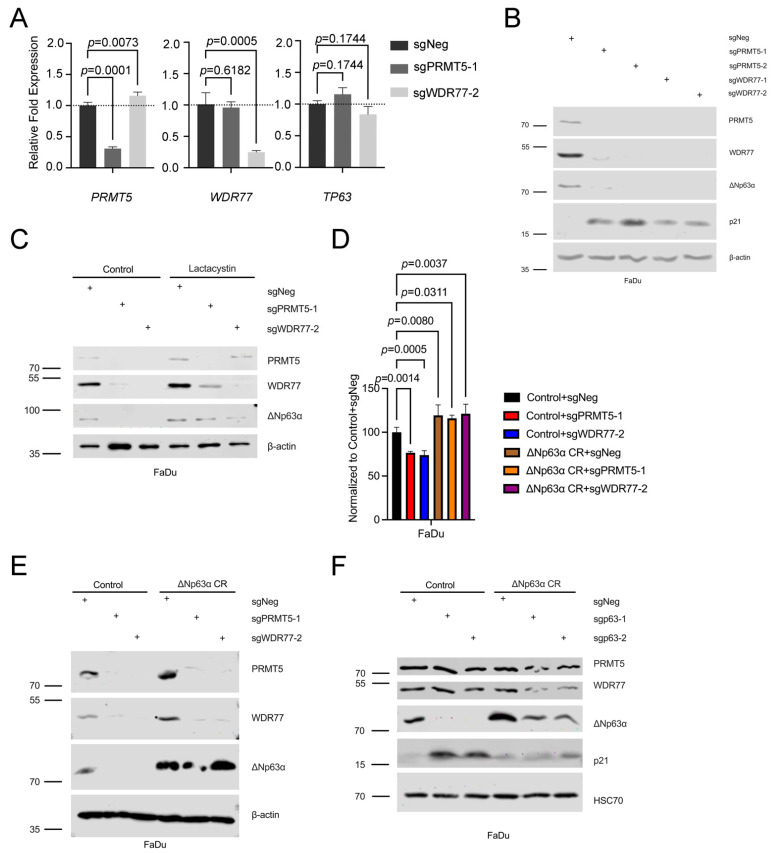
PRMT5 and WDR77 modulate the ΔNp63α-p21 pathway. (**A**) qRT-PCR for *PRMT5*, *WDR77* and *TP63* transcript levels in FaDu cells transduced with sgPRMT5-1 and sgWDR77-2, normalized to *GAPDH*. The *p*-values were calculated using One-Way ANOVA. (**B**) Western blot of PRMT5, WDR77, ΔNp63α, and p21 expression in FaDu cells transduced with sgNeg (empty vector control), sgPRMT5-1, sgPRMT5-2, sgWDR77-1, and sgWDR77-2. (**C**) FaDu cells were infected with sgNeg, sgPRMT5-1, and sgWDR77-2, with or without additional treatment of 1 μmol/L lactacystin for 24 h, as described [36]. (**D**) MTT-based proliferation assays in FaDu scramble cells (control) and FaDu cells overexpressing CRISPR-resistant ΔNp63α cDNAs (ΔNp63α CR). Cells were infected with sgNeg, sgPRMT5-1, and sgWDR77-2. Data are presented as means ± S.D., normalized to sgNeg (n = 3 biologically independent samples). The *p*-values were calculated using One-Way ANOVA. (**E**) Confirmation of PRMT5KO and WDR77KO via Western blot in FaDu scramble cells (control) and FaDu cells overexpressing ΔNp63α CR. Cells were treated with sgNeg, sgPRMT5-1, and sgWDR77-2. (**F**) Assessment of PRMT5, WDR77, ΔNp63α, and p21 expression via Western blot in FaDu scramble cells (control) and FaDu cells overexpressing ΔNp63α CR. Cells were treated with sgNeg (empty vector control), sgp63-1, and sgp63-2. The uncropped bolts were shown in Supplementary Figure S7.

To elucidate the genetic mechanism by which PRMT5/WDR77 depletion inhibits proliferation in SCC, we first performed qRT-PCR and found that neither loss of *PRMT5* nor *WDR77* significantly altered *TP63* transcript levels (Figure 4A, Supplementary Figure S5A and see Supplementary Figure S2A). Additionally, qRT-PCR validated the knockout efficacy in the HSC-5 cell samples used for RNA-seq (Supplementary Figure S5A). However, at the protein level, we observed that depletion of PRMT5 not only reduced PRMT5 protein as expected, it also led to the loss of WDR77; the same was also true in that depletion of WDR77 reduced PRMT5 protein, indicating their interdependence. Furthermore, depletion of either PRMT5 or WDR77 resulted in the downregulation of ΔNp63α and the simultaneous upregulation of p21 at the protein level (Figure 4B, Supplementary Figure S5B). As described above, whereas overexpression of WDR77 efficiently rescued the effect of WDR77 depletion, it failed to rescue the effect of PRMT5 loss (see Supplementary Figure S4C). Furthermore, depletion of either PRMT5 or WDR77 alone reduced cell proliferation (see Figure 3B and Supplementary Figure S4A).

Based on these observations, we hypothesized that the interdependency of PRMT5 and WDR77 was due to protein instability, and therefore, treated PRMT5KO and WDR77KO cells with the proteasome inhibitor lactacystin [35]. Indeed, lactacystin effectively rescued both WDR77 and ΔNp63α expression in the context of PRMT5 depletion; lactacystin also effectively rescued both PRMT5 and ΔNp63α expression in the context of WDR77 depletion (Figure 4C, Supplementary Figure S5C). This indicated that PRMT5 impacted WDR77 by preventing its protein degradation and vice versa, which also extended to the downstream target protein ΔNp63α. Reduced cell proliferation due to PRMT5 or WDR77 depletion can be rescued by ΔNp63α expression (Figure 4D, Supplementary Figure S5D). Additionally, the expression of PRMT5 or WDR77 was independent of ΔNp63α overexpression (Figure 4E, Supplementary Figure S5E). We also show that p21 was upregulated upon loss of ΔNp63α, an effect rescued by ΔNp63α expression (Figure 4F, Supplementary Figure S5F). These findings demonstrate that PRMT5 and WDR77 are each essential for the stability of the other and establish ΔNp63α-mediated regulation of p21 as part of the mechanism.

### 3.5. PRMT5 and WDR77 Depletion Repress SCC In Vivo

**Figure 5:**
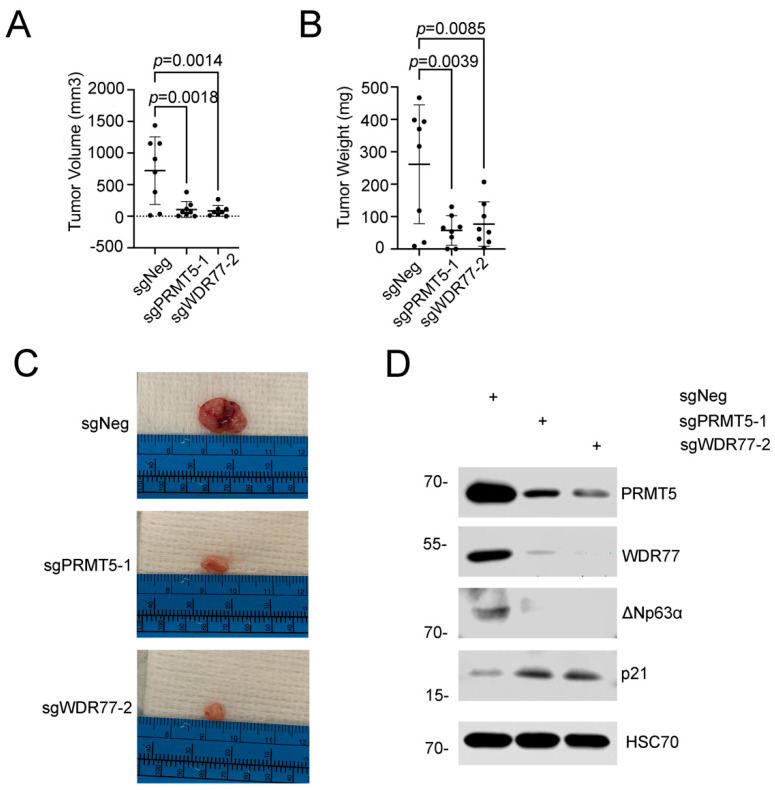
PRMT5 or WDR77 depletion inhibits tumor growth in vivo. (**A**,**B**) Tumor volume caliper measurements and tumor weight measurements are means ± S.D. (n = 8). FaDu cells transduced with sgNeg, sgPRMT5-1, and sgWDR77-2 were injected subcutaneously into both rear flanks of nude mice (n = 4), with 5 × 10^4^ cells per injection. Tumors were measured, weighed, and collected after 22 days. Mice were initially labeled as groups 1, 2, and 3, with the treatment groups blinded to ensure unbiased measurements until all data collection was completed. The *p*-values were calculated using One-Way ANOVA. (**C**) Representative images of tumors from each group. (**D**) Western blot assessment of PRMT5, WDR77, ΔNp63α, and p21 expression in the tumor samples from mice. The uncropped bolts were shown in Supplementary Figure S7.

To further determine the effect of depleting PRMT5 and WDR77 in SCC development in the in vivo context, we injected 5 × 10^4^ sgNeg, sgPRMT5, and sgWDR77 cells subcutaneously into nude mice to generate tumor xenografts. We observed a significant reduction in tumor size and weight in the KO groups compared to the control (Figure 5A–C). Depletion of PRMT5 and WDR77 resulted in downregulation of ΔNp63α and upregulation of p21 in vivo, consistent with our findings in cultured SCC cells (Figure 5D and Supplementary Figure S6A). These findings indicate that depletion of either PRMT5 or WDR77 impairs SCC development in vivo.

## 4. Discussion

Epigenetic dysregulation is a common feature among cancers. This dysregulation often results in increased expression of oncogenes, or inactivation of tumor suppressor genes. The reversible nature of the epigenetic state makes chromatin modifiers valuable drug targets, particularly in SCC, where little progress has been made in the development of targeted therapies [1,37]. Here, we identify the methyltransferase PRMT5 as essential to SCC proliferation. The canonical function of PRMT5 is to catalyze the symmetric dimethylation of H4R3me2s and H3R8me2s, leading to chromatin compaction and gene silencing [5]. This activity is enhanced by the formation of an octameric complex consisting of four PRMT5 and four WDR77 subunits [8]. PRMT5 and WDR77 are upregulated in a number of human cancers, including breast cancer [38], pancreatic cancer [39], glioblastoma [40], and lung cancer [41], and associated with poor survival, underscoring their essential role in oncogenesis [42]. A key reason for the enhanced PRMT5 expression seen in cancers is the ability of PRMT5 to facilitate cell cycle progression. While previous studies have identified a role of PRMT5 in the regulation of TRIM12/TXNIP [43], PTEN [44], p53 [45,46], and p21 [47], there is to date no data linking PRMT5 to ΔNp63α, a critical regulator of proliferation and differentiation. We have addressed this problem by showing that ΔNp63α is a downstream target of PRMT5. In the current study, we focused on the genetic mechanism that was caused by the silencing of both PRMT5 and WDR77. Herein, we show that PRMT5 and WDR77 are elevated in SCC, and essential for SCC proliferation. This is in line with previous work showing that depletion of PRMT5 and WDR77 in epidermal keratinocytes compromises cell proliferation and enhances differentiation [47,48]. These findings parallel our previous work with ΔNp63α, which showed that ΔNp63α is frequently overexpressed and maintains a proliferative and undifferentiated phenotype in SCC [15,18]. Because of this, we set out to determine whether there was a link between PRMT5 and ΔNp63α expression.

While PRMT5 has been previously linked to TAp63 [49,50], regulation of ΔNp63 isoforms by PRMT5 has not been established. In SCC, ΔNp63α is the predominant isoform and often plays an opposing role to TAp63. TAp63 contains the N-terminal transactivation domain and is structurally similar to p53 [51], acting as a tumor suppressor [52]. In contrast, ΔNp63 isoforms lack the N-terminal transactivation domain and act as oncogenes [53]. Given the close relationship between TAp63 and ΔNp63, it was crucial to explore the potential interaction between PRMT5 and ΔNp63α, especially in SCC, where ΔNp63α, rather than TAp63, is predominantly expressed. Our findings highlight a unique role for PRMT5 in regulating ΔNp63α in SCC, distinct from the established PRMT5/TAp63 interaction in other cancers, offering new insights into SCC biology.

To our knowledge, this study is the first to explore the connection between PRMT5 and ΔNp63α in the context of HPV-negative HNSCC and CSCC. Head and neck squamous cell carcinomas (HNSCC) caused by tobacco usage have a higher mutation burden compared to HPV-positive tumors [54]. Among these mutations, loss-of-function mutations of p53 are one of the most prevalent in HPV-negative HNSCC [55] and CSCC [56]. Considering that both of these two SCCs bear a significant burden of p53 loss-of-function mutations [2,55,56], this underscored the need to investigate mechanisms that operate independently of p53 activation in the context of HPV-negative HNSCC and CSCC. Therefore, although both ΔNp63α and PRMT5 have been linked to p53 [10,45], we utilized three cell lines, all of which possess inactivating p53 mutations [57,58,59], to eliminate the influence of functional p53. It is noteworthy that ΔNp63α can regulate p21 through both p53-dependent [12] and p53-independent pathways [13]. Furthermore, ΔNp63α has been shown to bind directly to the promoter region of p21 [12,53,60], highlighting the complexity and significance of our findings. Herein, we provide evidence that PRMT5 regulates p21 expression independent of functional p53, via the regulation of ΔNp63α.

Finally, we demonstrate that PRMT5 stabilization of ΔNp63α occurs at the protein level. One plausible hypothesis is that PRMT5 regulates specific E3 ubiquitin ligases involved in p63 protein degradation [61]. PRMT5 has previously been shown to regulate the expression of ITCH (Itchy E3 ubiquitin ligase) [62], which promotes the degradation of ΔNp63α in keratinocytes [63]. Additionally, PRMT5 can suppress the E3 ubiquitin ligase FBXW7 (F-box and WD-40 domain-containing protein 7, also known as FBW7) in pancreatic cancer [64]. In this context, suppression of PRMT5 led to an increase in FBXW7, resulting in reduced c-MYC protein despite stable c-MYC mRNA expression. FBXW7 has also been linked to ΔNp63α protein degradation, making this another potential mechanism [65]. Our RNA-seq data indicated that *ITCH* and *FBXO30,* which encode a separate ubiquitin ligase not previously linked to either PRMT5 or ΔNp63α, exhibited significant changes in response to *PRMT5* and *WDR77* silencing, offering valuable preliminary insights. Future work will focus on the precise mediator of PRMT5 regulation of ΔNp63α protein expression.

This work has important therapeutic implications. Notably, the selective PRMT5 inhibitor, PF-06939999, is in a phase I clinical trial for HNSCC patients [66]. Identifying the direct downstream targets of PRMT5 and WDR77 provides avenues for expanding the utility of PRMT5 inhibitors such as PF-06939999. This is especially important for SCC patients where PRMT5 and ΔNp63α are both upregulated and contribute to enhanced proliferation and the maintenance of an undifferentiated phenotype. Thus, the PRMT5/WDR77-ΔNp63α-p21 axis has potential as a therapy target in SCC patients to suppress proliferation and produce a more differentiated phenotype.

## 5. Conclusions

In summary, this study highlights that PRMT5 is overexpressed in squamous cell carcinoma (SCC) and correlates with poor patient survival. Our mechanistic investigations reveal that both PRMT5 and its binding partner WDR77 are critical for SCC survival, primarily by regulating cell proliferation. This regulation is achieved through increased ΔNp63α protein expression, which in turn suppresses p21 expression. These findings offer novel insights into potential treatment strategies for SCC, emphasizing the therapeutic value of targeting the PRMT5/WDR77-ΔNp63α-p21 axis.

## Data Availability

The RNA-seq data generated in this study are available in the GEO database with accession number GSE272319. The single-cell RNA-seq data are from dataset GSE103322 in the GEO database.

## Supplementary Materials

The following supporting information can be downloaded at: https://www.mdpi.com/article/10.3390/cancers16223789/s1. Table S1: sgRNAs and PCR primer sequences; Figure S1: Analyses of public datasets reveal that *PRMT5* is linked to poor SCC survival, and gene editing reveals that PRMT5 is essential to cancer cells; Figure S2: RNA-seq data analysis; Figure S3: Single-cell RNA-seq data analysis; Figure S4: Supportive data for negative selection assays and flow cytometry assays; Figure S5: PRMT5 and WDR77 modulates the ΔNp63α-p21 pathway in SCC cell lines; Figure S6: PRMT5 and WDR77 modulates the ΔNp63α-p21 pathway in vivo; Figure S7: Original Western blot images; Figure S8: Original images of tumors from mice.

## References

[1] Johnson D.E., Burtness B., Leemans C.R., Lui V.W.Y., Bauman J.E., Grandis J.R. (2020). Head and neck squamous cell carcinoma. Nat. Rev. Dis. Primers.

[2] Dotto G.P., Rustgi A.K. (2016). Squamous Cell Cancers: A Unified Perspective on Biology and Genetics. Cancer Cell.

[3] Ang K.K., Harris J., Wheeler R., Weber R., Rosenthal D.I., Nguyen-Tân P.F., Westra W.H., Chung C.H., Jordan R.C., Lu C. (2010). Human papillomavirus and survival of patients with oropharyngeal cancer. N. Engl. J. Med..

[4] Sánchez-Danés A., Blanpain C. (2018). Deciphering the cells of origin of squamous cell carcinomas. Nat. Rev. Cancer.

[5] Copeland R.A., Solomon M.E., Richon V.M. (2009). Protein methyltransferases as a target class for drug discovery. Nat. Rev. Drug Discov..

[6] Yang Y., Bedford M.T. (2013). Protein arginine methyltransferases and cancer. Nat. Rev. Cancer.

[7] Burgos E.S., Wilczek C., Onikubo T., Bonanno J.B., Jansong J., Reimer U., Shechter D. (2015). Histone H2A and H4 N-terminal tails are positioned by the MEP50 WD repeat protein for efficient methylation by the PRMT5 arginine methyltransferase. J. Biol. Chem..

[8] Antonysamy S., Bonday Z., Campbell R.M., Doyle B., Druzina Z., Gheyi T., Han B., Jungheim L.N., Qian Y., Rauch C. (2012). Crystal structure of the human PRMT5:MEP50 complex. Proc. Natl. Acad. Sci. USA.

[9] Fisher M.L., Balinth S., Mills A.A. (2020). p63-related signaling at a glance. J. Cell Sci..

[10] Keyes W.M., Mills A.A. (2006). p63: A new link between senescence and aging. Cell Cycle.

[11] Candi E., Cipollone R., Rivetti di Val Cervo P., Gonfloni S., Melino G., Knight R. (2008). p63 in epithelial development. Cell. Mol. Life Sci..

[12] Westfall M.D., Mays D.J., Sniezek J.C., Pietenpol J.A. (2003). The Delta Np63 alpha phosphoprotein binds the p21 and 14-3-3 sigma promoters in vivo and has transcriptional repressor activity that is reduced by Hay-Wells syndrome-derived mutations. Mol. Cell. Biol..

[13] Rocco J.W., Leong C.O., Kuperwasser N., DeYoung M.P., Ellisen L.W. (2006). p63 mediates survival in squamous cell carcinoma by suppression of p73-dependent apoptosis. Cancer Cell.

[14] Hozumi Y., Kondo S., Shimoura T., Aso K. (1990). Human squamous cell carcinoma from skin: Establishment and characterization of a new cell line (HSC-5). J. Dermatol..

[15] Fisher M.L., Balinth S., Hwangbo Y., Wu C., Ballon C., Wilkinson J.E., Goldberg G.L., Mills A.A. (2021). BRD4 Regulates Transcription Factor ΔNp63α to Drive a Cancer Stem Cell Phenotype in Squamous Cell Carcinomas. Cancer Res..

[16] Team R.C. (2024). R: A Language and Environment for Statistical Computing.

[17] Sun X., Klingbeil O., Lu B., Wu C., Ballon C., Ouyang M., Wu X.S., Jin Y., Hwangbo Y., Huang Y.H. (2023). BRD8 maintains glioblastoma by epigenetic reprogramming of the p53 network. Nature.

[18] Balinth S., Fisher M.L., Hwangbo Y., Wu C., Ballon C., Sun X., Mills A.A. (2022). EZH2 regulates a SETDB1/ΔNp63α axis via RUNX3 to drive a cancer stem cell phenotype in squamous cell carcinoma. Oncogene.

[19] Shi J., Wang E., Milazzo J.P., Wang Z., Kinney J.B., Vakoc C.R. (2015). Discovery of cancer drug targets by CRISPR-Cas9 screening of protein domains. Nat. Biotechnol..

[20] Dobin A., Davis C.A., Schlesinger F., Drenkow J., Zaleski C., Jha S., Batut P., Chaisson M., Gingeras T.R. (2013). STAR: Ultrafast universal RNA-seq aligner. Bioinformatics.

[21] Anders S., Pyl P.T., Huber W. (2015). HTSeq—A Python framework to work with high-throughput sequencing data. Bioinformatics.

[22] Love M.I., Huber W., Anders S. (2014). Moderated estimation of fold change and dispersion for RNA-seq data with DESeq2. Genome Biol..

[23] Subramanian A., Tamayo P., Mootha V.K., Mukherjee S., Ebert B.L., Gillette M.A., Paulovich A., Pomeroy S.L., Golub T.R., Lander E.S. (2005). Gene set enrichment analysis: A knowledge-based approach for interpreting genome-wide expression profiles. Proc. Natl. Acad. Sci. USA.

[24] Korotkevich G., Sukhov V., Budin N., Shpak B., Artyomov M.N., Sergushichev A. (2021). Fast gene set enrichment analysis. bioRxiv.

[25] Wickham H., François R., Henry L., Müller K., Vaughan D. (2023). dplyr: A Grammar of Data Manipulation.

[26] Wickham H. (2016). ggplot2: Elegant Graphics for Data Analysis.

[27] Hao Y., Stuart T., Kowalski M.H., Choudhary S., Hoffman P., Hartman A., Srivastava A., Molla G., Madad S., Fernandez-Granda C. (2024). Dictionary learning for integrative, multimodal and scalable single-cell analysis. Nat. Biotechnol..

[28] Tanida-Miyake E., Koike M., Uchiyama Y., Tanida I. (2018). Optimization of mNeonGreen for Homo sapiens increases its fluorescent intensity in mammalian cells. PLoS ONE.

[29] Kassambara A. Survminer: Survival Analysis and Visualization. https://github.com/kassambara/survminer.

[30] Liberzon A., Subramanian A., Pinchback R., Thorvaldsdóttir H., Tamayo P., Mesirov J.P. (2011). Molecular signatures database (MSigDB) 3.0. Bioinformatics.

[31] Liberzon A., Birger C., Thorvaldsdóttir H., Ghandi M., Mesirov J.P., Tamayo P. (2015). The Molecular Signatures Database (MSigDB) hallmark gene set collection. Cell Syst..

[32] Rickman D.S., Millon R., De Reynies A., Thomas E., Wasylyk C., Muller D., Abecassis J., Wasylyk B. (2008). Prediction of future metastasis and molecular characterization of head and neck squamous-cell carcinoma based on transcriptome and genome analysis by microarrays. Oncogene.

[33] Puram S.V., Tirosh I., Parikh A.S., Patel A.P., Yizhak K., Gillespie S., Rodman C., Luo C.L., Mroz E.A., Emerick K.S. (2017). Single-Cell Transcriptomic Analysis of Primary and Metastatic Tumor Ecosystems in Head and Neck Cancer. Cell.

[34] Jensen-Pergakes K., Tatlock J., Maegley K.A., McAlpine I.J., McTigue M., Xie T., Dillon C.P., Wang Y., Yamazaki S., Spiegel N. (2022). SAM-Competitive PRMT5 Inhibitor PF-06939999 Demonstrates Antitumor Activity in Splicing Dysregulated NSCLC with Decreased Liability of Drug Resistance. Mol. Cancer Ther..

[35] Ōmura S., Crump A. (2019). Lactacystin: First-in-class proteasome inhibitor still excelling and an exemplar for future antibiotic research. J. Antibiot..

[36] Koster S., Gurumurthy R.K., Kumar N., Prakash P.G., Dhanraj J., Bayer S., Berger H., Kurian S.M., Drabkina M., Mollenkopf H.J. (2022). Modelling Chlamydia and HPV co-infection in patient-derived ectocervix organoids reveals distinct cellular reprogramming. Nat. Commun..

[37] Mesgari H., Esmaelian S., Nasiri K., Ghasemzadeh S., Doroudgar P., Payandeh Z. (2023). Epigenetic Regulation in Oral Squamous Cell Carcinoma Microenvironment: A Comprehensive Review. Cancers.

[38] Chiang K., Zielinska A.E., Shaaban A.M., Sanchez-Bailon M.P., Jarrold J., Clarke T.L., Zhang J., Francis A., Jones L.J., Smith S. (2017). PRMT5 Is a Critical Regulator of Breast Cancer Stem Cell Function via Histone Methylation and FOXP1 Expression. Cell Rep..

[39] Ge L., Wang H., Xu X., Zhou Z., He J., Peng W., Du F., Zhang Y., Gong A., Xu M. (2020). PRMT5 promotes epithelial-mesenchymal transition via EGFR-β-catenin axis in pancreatic cancer cells. J. Cell. Mol. Med..

[40] Yan F., Alinari L., Lustberg M.E., Martin L.K., Cordero-Nieves H.M., Banasavadi-Siddegowda Y., Virk S., Barnholtz-Sloan J., Bell E.H., Wojton J. (2014). Genetic validation of the protein arginine methyltransferase PRMT5 as a candidate therapeutic target in glioblastoma. Cancer Res..

[41] Zhang S., Ma Y., Hu X., Zheng Y., Chen X. (2019). Targeting PRMT5/Akt signalling axis prevents human lung cancer cell growth. J. Cell. Mol. Med..

[42] Stopa N., Krebs J.E., Shechter D. (2015). The PRMT5 arginine methyltransferase: Many roles in development, cancer and beyond. Cell. Mol. Life Sci..

[43] Li Y.H., Tong K.L., Lu J.L., Lin J.B., Li Z.Y., Sang Y., Ghodbane A., Gao X.J., Tam M.S., Hu C.D. (2020). PRMT5-TRIM21 interaction regulates the senescence of osteosarcoma cells by targeting the TXNIP/p21 axis. Aging (Albany NY).

[44] Banasavadi-Siddegowda Y.K., Russell L., Frair E., Karkhanis V.A., Relation T., Yoo J.Y., Zhang J., Sif S., Imitola J., Baiocchi R. (2017). PRMT5-PTEN molecular pathway regulates senescence and self-renewal of primary glioblastoma neurosphere cells. Oncogene.

[45] Jansson M., Durant S.T., Cho E.C., Sheahan S., Edelmann M., Kessler B., La Thangue N.B. (2008). Arginine methylation regulates the p53 response. Nat. Cell Biol..

[46] Che Y., Liu Y., Yao Y., Hill H.A., Li Y., Cai Q., Yan F., Jain P., Wang W., Rui L. (2023). Exploiting PRMT5 as a target for combination therapy in mantle cell lymphoma characterized by frequent ATM and TP53 mutations. Blood Cancer J..

[47] Saha K., Eckert R.L. (2015). Methylosome Protein 50 and PKCδ/p38δ Protein Signaling Control Keratinocyte Proliferation via Opposing Effects on p21Cip1 Gene Expression. J. Biol. Chem..

[48] Kanade S.R., Eckert R.L. (2012). Protein arginine methyltransferase 5 (PRMT5) signaling suppresses protein kinase Cδ- and p38δ-dependent signaling and keratinocyte differentiation. J. Biol. Chem..

[49] Liu M., Yao B., Gui T., Guo C., Wu X., Li J., Ma L., Deng Y., Xu P., Wang Y. (2020). PRMT5-dependent transcriptional repression of c-Myc target genes promotes gastric cancer progression. Theranostics.

[50] Yabuta N., Ota C., Sasakura T., Naito Y., Okuzaki D., Fukushima K., Nojima H. (2018). Late cornified envelope 1C (LCE1C), a transcriptional target of TAp63 phosphorylated at T46/T281, interacts with PRMT5. Sci. Rep..

[51] Murray-Zmijewski F., Lane D.P., Bourdon J.C. (2006). p53/p63/p73 isoforms: An orchestra of isoforms to harmonise cell differentiation and response to stress. Cell Death Differ..

[52] Su X., Chakravarti D., Cho M.S., Liu L., Gi Y.J., Lin Y.L., Leung M.L., El-Naggar A., Creighton C.J., Suraokar M.B. (2010). TAp63 suppresses metastasis through coordinate regulation of Dicer and miRNAs. Nature.

[53] Keyes W.M., Pecoraro M., Aranda V., Vernersson-Lindahl E., Li W., Vogel H., Guo X., Garcia E.L., Michurina T.V., Enikolopov G. (2011). ΔNp63α is an oncogene that targets chromatin remodeler Lsh to drive skin stem cell proliferation and tumorigenesis. Cell Stem Cell.

[54] Agrawal N., Frederick M.J., Pickering C.R., Bettegowda C., Chang K., Li R.J., Fakhry C., Xie T.X., Zhang J., Wang J. (2011). Exome sequencing of head and neck squamous cell carcinoma reveals inactivating mutations in NOTCH1. Science.

[55] (2015). Comprehensive genomic characterization of head and neck squamous cell carcinomas. Nature.

[56] Inman G.J., Wang J., Nagano A., Alexandrov L.B., Purdie K.J., Taylor R.G., Sherwood V., Thomson J., Hogan S., Spender L.C. (2018). The genomic landscape of cutaneous SCC reveals drivers and a novel azathioprine associated mutational signature. Nat. Commun..

[57] Hoque M.O., Begum S., Sommer M., Lee T., Trink B., Ratovitski E., Sidransky D. (2003). PUMA in head and neck cancer. Cancer Lett..

[58] Tanaka H., Shibagaki I., Shimada Y., Wagata T., Imamura M., Ishizaki K. (1996). Characterization of p53 gene mutations in esophageal squamous cell carcinoma cell lines: Increased frequency and different spectrum of mutations from primary tumors. Int. J. Cancer.

[59] Magné N., Fischel J.L., Dubreuil A., Formento P., Poupon M.F., Laurent-Puig P., Milano G. (2002). Influence of epidermal growth factor receptor (EGFR), p53 and intrinsic MAP kinase pathway status of tumour cells on the antiproliferative effect of ZD1839 (“Iressa”). Br. J. Cancer.

[60] Yang A., Zhu Z., Kapranov P., McKeon F., Church G.M., Gingeras T.R., Struhl K. (2006). Relationships between p63 binding, DNA sequence, transcription activity, and biological function in human cells. Mol. Cell.

[61] Li C., Xiao Z.X. (2014). Regulation of p63 protein stability via ubiquitin-proteasome pathway. Biomed Res. Int..

[62] Borbora S.M., Rajmani R.S., Balaji K.N. (2022). PRMT5 epigenetically regulates the E3 ubiquitin ligase ITCH to influence lipid accumulation during mycobacterial infection. PLoS Pathog..

[63] Rossi M., Aqeilan R.I., Neale M., Candi E., Salomoni P., Knight R.A., Croce C.M., Melino G. (2006). The E3 ubiquitin ligase Itch controls the protein stability of p63. Proc. Natl. Acad. Sci. USA.

[64] Qin Y., Hu Q., Xu J., Ji S., Dai W., Liu W., Xu W., Sun Q., Zhang Z., Ni Q. (2019). PRMT5 enhances tumorigenicity and glycolysis in pancreatic cancer via the FBW7/cMyc axis. Cell Commun. Signal..

[65] Galli F., Rossi M., D’Alessandra Y., De Simone M., Lopardo T., Haupt Y., Alsheich-Bartok O., Anzi S., Shaulian E., Calabrò V. (2010). MDM2 and Fbw7 cooperate to induce p63 protein degradation following DNA damage and cell differentiation. J. Cell Sci..

[66] Rodon J., Rodriguez E., Maitland M.L., Tsai F.Y., Socinski M.A., Berlin J.D., Thomas J.S., Al Baghdadi T., Wang I.M., Guo C. (2024). A phase I study to evaluate the safety, pharmacokinetics, and pharmacodynamics of PF-06939999 (PRMT5 inhibitor) in patients with selected advanced or metastatic tumors with high incidence of splicing factor gene mutations. ESMO Open.

