# Supplementary material for: PRMT5/WDR77 Enhances the Proliferation of Squamous Cell Carcinoma via the ΔNp63α-p21 Axis
Source: Cancers (Basel). 2024 Nov 11;16(22):3789. doi: 10.3390/cancers16223789 (PMC11592282; doi:10.3390/cancers16223789)

PRMT5/WDR77 Enhance Proliferation of Squamous Cell Carcinoma via the ΔNp63α-p21 Axis

Supplementary Materials

Table S1. sgRNAs and PCR primer sequences.

PCR Primers	Sequence (5′ –> 3′)
PRMT5	Forward: TAGCTGACACACTAGGGGCT<br>Reverse: GGGCCGATTCTTAGCAGGTT
WDR77	Forward: CGGCTCCCTCTGGCTTTTTA<br>Reverse: AACAGCACCTGAATCGGAGG
TP63	Forward: CCTGGAAAACAATGCCCAGAC<br>Reverse: ACGAGGAGCCGTTCTGAATC
GAPDH	Forward: AATCCCATCACCATCTTCCA<br>Reverse: TGGACTCCACGACGTACTCA
**sgRNAs**	**Sequence (5′ –> 3′)**
sgPRMT5-1	CAGCATACAGCTTTATCCGC
sgPRMT5-2	CTGATGAGACTACGGTCACT
sgWDR77-1	ATGCGCCCGCCTGCATGGAA
sgWDR77-2	CTCCGCCGGAGTCCAAACGG
sgp63-1	CCCTCCAACACCGACTACCC
sgp63-2	GCCTGGGTAGTCGGTGTTGG

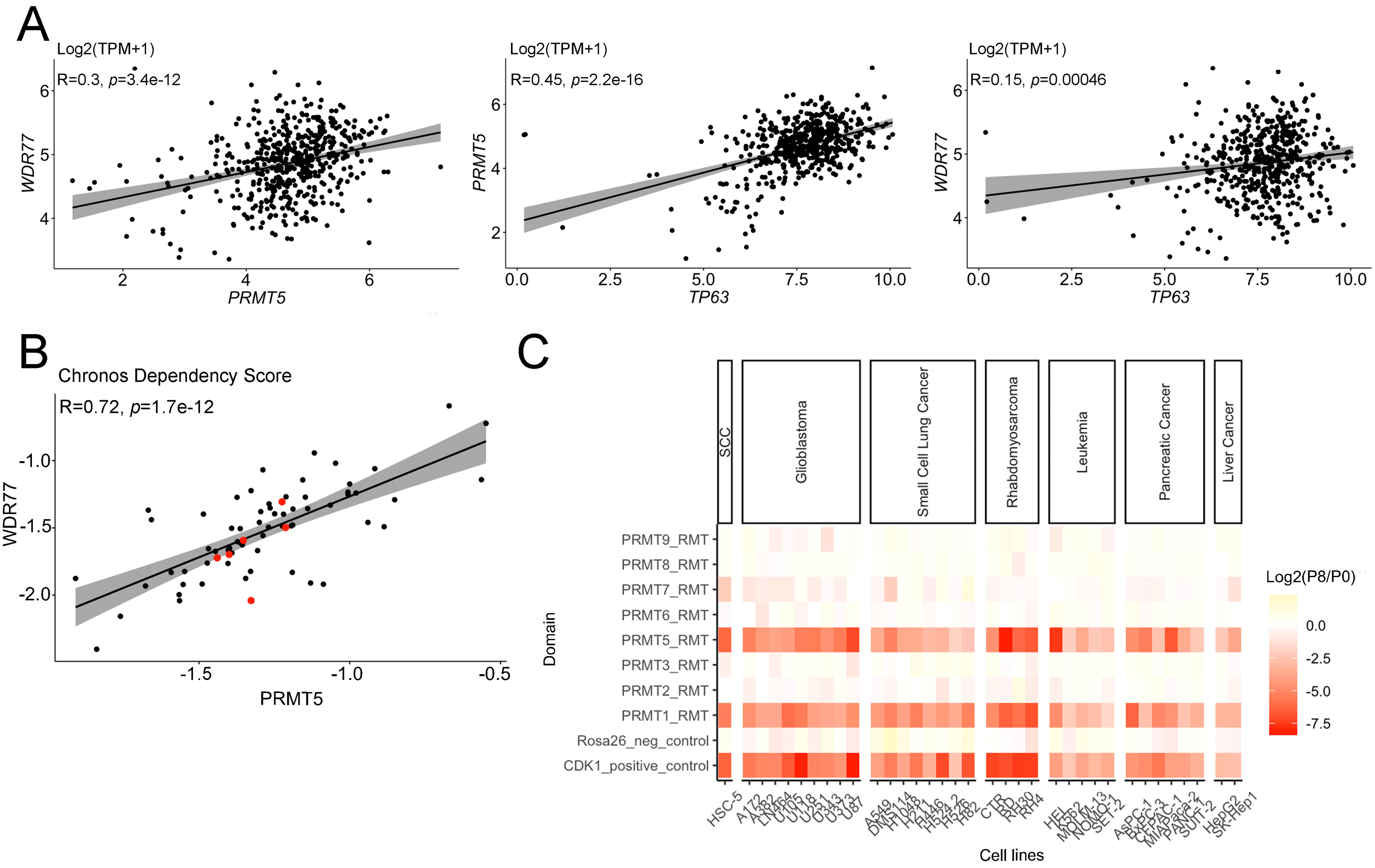

Figure S1. Analyses of public datasets reveal that *PRMT5* is linked to poor SCC survival, and gene editing reveals PRMT5 is essential to cancer cells. (A) Gene expression correlation analysis between *PRMT5*, *TP63* and *WDR77* in TCGA-HNSC (n = 513) using Pearson Correlation Test. (B) Correlation

Analysis of DepMap Data including diverse cancer cell lines, with emphasis on six squamous carcinoma lines (SCC lines highlighted in red). (C) Domain-focused CRISPR screening revealed *PRMT5* to be essential in multiple cancers including SCC.

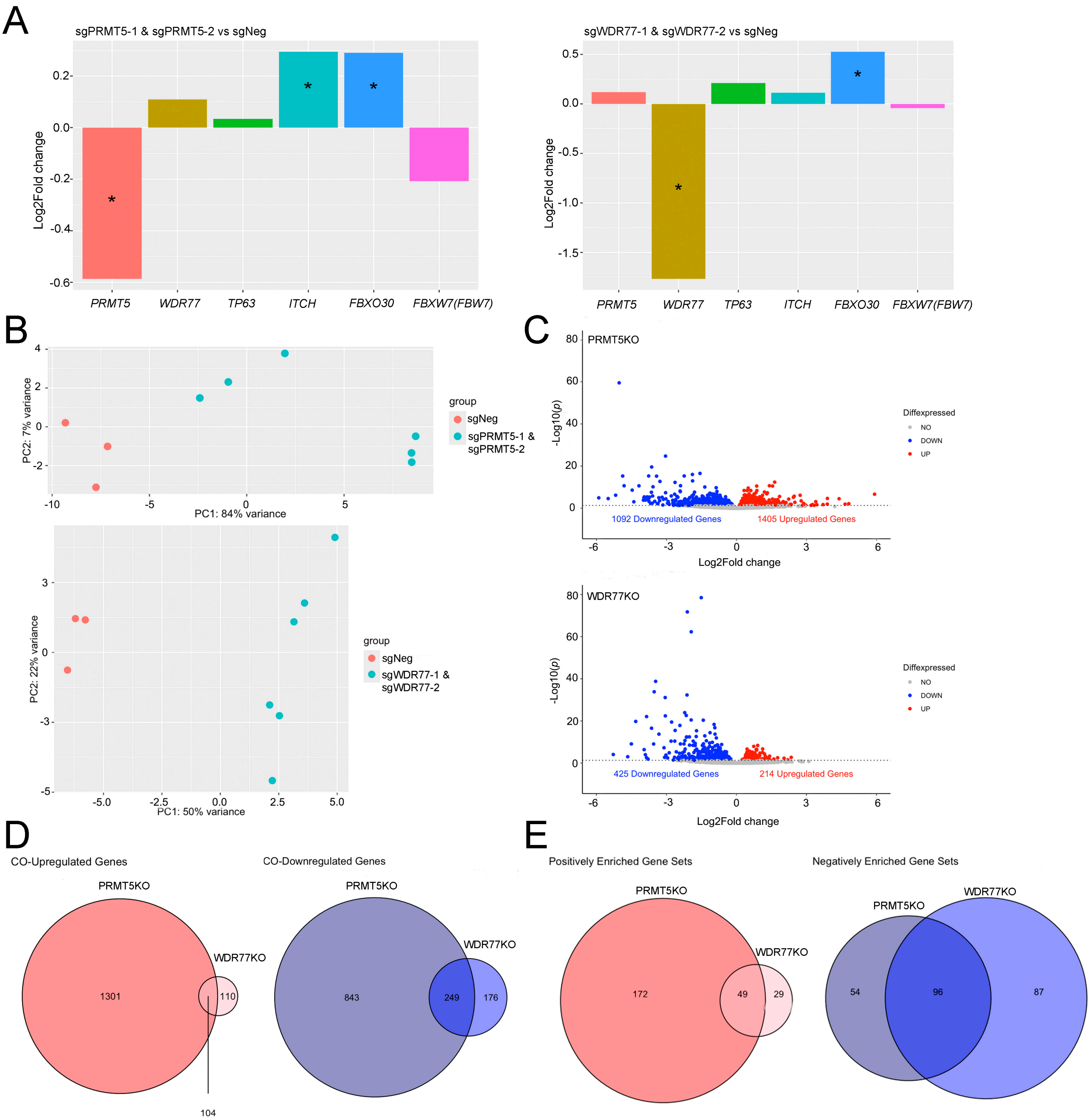

Upregulated genes	WDR77KO	Non_WDR77KO
PRMT5KO	104	1301
Non_PRMT5KO	110	12241

p=6.219168e-47
Odds ratio=8.892885

Downregulated genes	WDR77KO	Non_WDR77KO
PRMT5KO	249	843
Non_PRMT5KO	176	12488

p=3.442725e-168
Odds ratio=20.93858

Positive enrichment	WDR77KO	Non_WDR77KO
PRMT5KO	49	172
Non_PRMT5KO	29	6245

p=4.218715e-54
Odds ratio=61.03884

Negative enrichment	WDR77KO	Non_WDR77KO
PRMT5KO	96	54
Non_PRMT5KO	87	6287

p=1.368869e-121
Odds ratio= 127.0456

Figure S2. RNA-seq data analysis. Bulk RNA-seq was performed on HSC5 cells treated under five different conditions: Control (sgNeg), sgPRMT5-1, sgPRMT5-2, sgWDR77-1, and sgWDR77-2, with each group consisting of three biological replicates. (A) Display of mRNA expression levels of *PRMT5*, *WDR77*, *TP63* and potential downstream targets in PRMT5KO (left) and WDR77KO (right) cells. (B) PCA plots. (C) Volcano plots. (D)Venn diagram illustration of the intersection of genes significantly upregulated (left, red) and downregulated (right, blue) by PRMT5KO and WDR77KO. Gene Differential expression of genes was computed using DeSeq2, with genes having a BaseMean less than 3 excluded. Contingency tables were constructed for respective groups, and Fisher's Exact Test was used to assess the relationship between the two knockout groups. (E) Venn diagram illustration of the intersection of gene sets positively enriched (left, red) and negatively enriched (right, blue) by PRMT5KO and WDR77KO from GSEA.

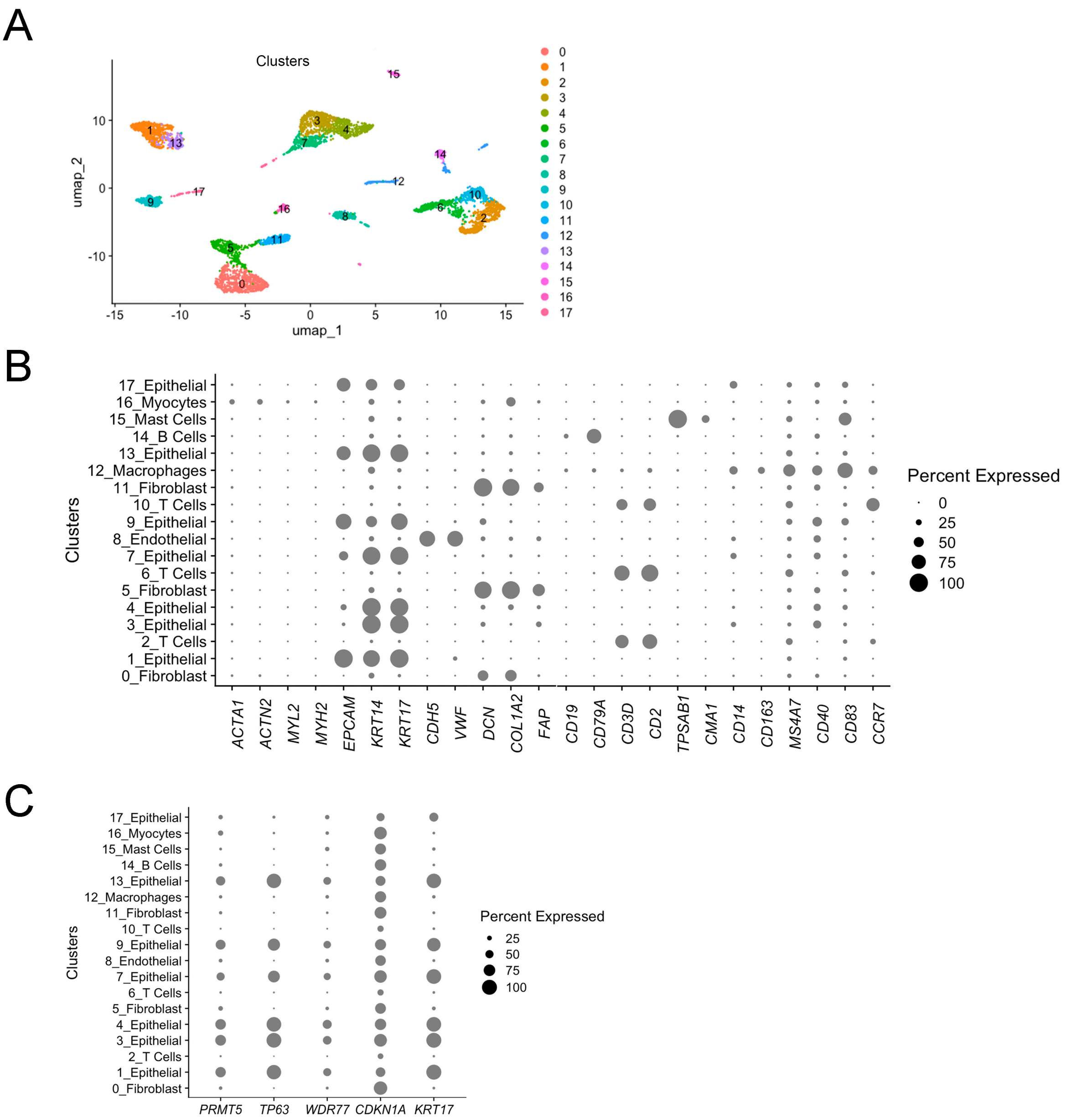

Figure S3. Single-cell RNA-seq data analysis. (A) UMAP plot of 5902 cells grouped in 18 clusters. (B) Feature plots illustrated canonical marker genes and their percentages of expression across clusters. (C) Feature plots illustrated genes including *PRMT5*, *TP63*, *WDR77*, *CDKN1A*, and *KRT17*, and their percentages of expression across clusters.

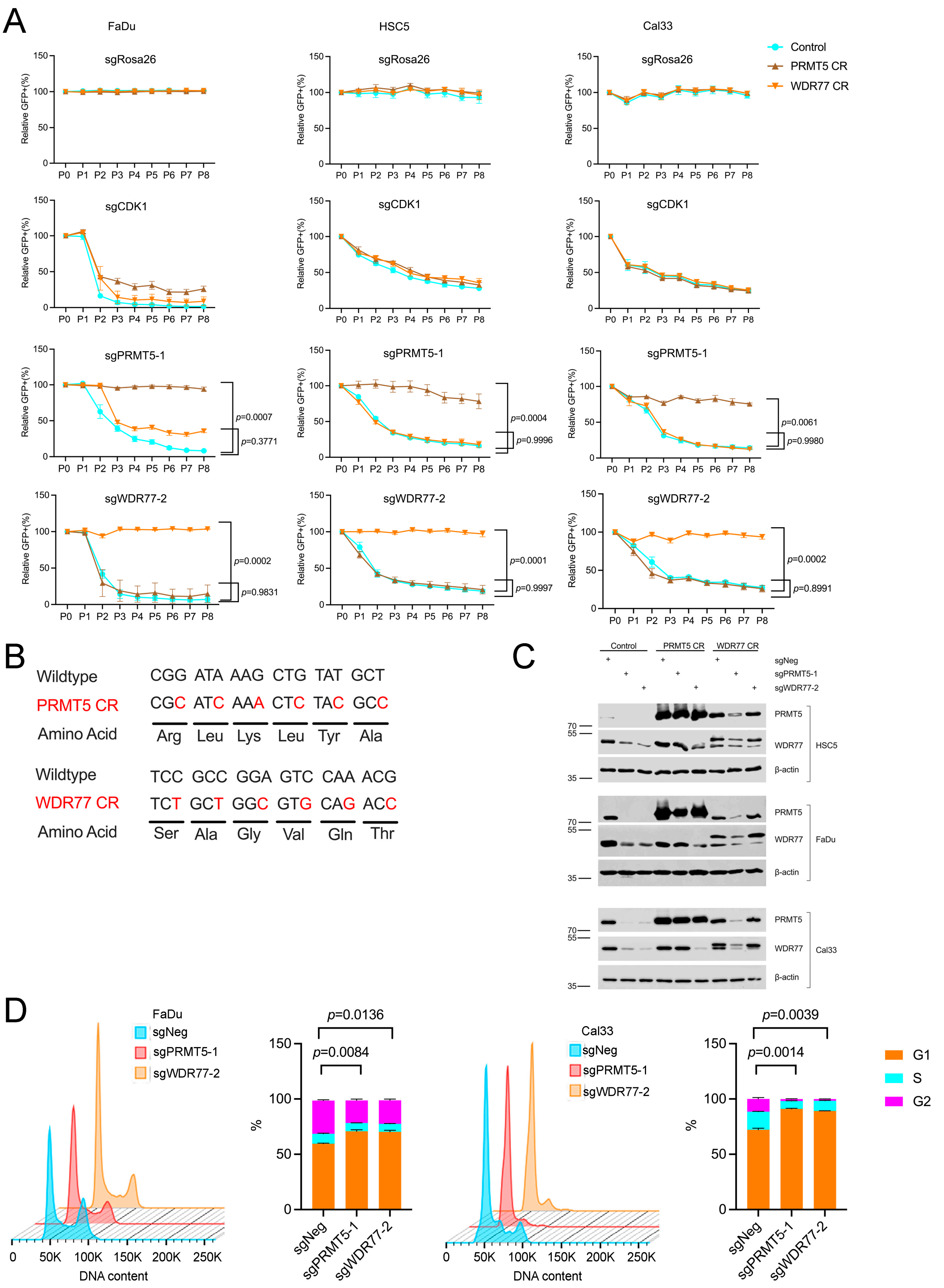
A
FaDu
HSC5
Cal33
Control
PRMT5 CR
WDR77 CR
sgRosa26
sgCDK1
sgPRMT5-1
sgWDR77-2
Relative GFP+(%)
P0 P1 P2 P3 P4 P5 P6 P7 P8
p=0.0007
p=0.3771
p=0.0004
p=0.9996
p=0.0061
p=0.9980
p=0.0002
p=0.9831
p=0.0001
p=0.9997
p=0.0002
p=0.8991
B
Wildtype CGG ATA AAG CTG TAT GCT
PRMT5 CR CGC ATC AAA CTC TAC GCC
Amino Acid Arg Leu Lys Leu Tyr Ala
Wildtype TCC GCC GGA GTC CAA ACG
WDR77 CR TCT GCT GGC GTG CAG ACC
Amino Acid Ser Ala Gly Val Gln Thr
C
Control
PRMT5 CR
WDR77 CR
sgNeg
sgPRMT5-1
sgWDR77-2
PRMT5
WDR77
β-actin
HSC5
FaDu
Cal33
70
55
35
D
FaDu
Cal33
sgNeg
sgPRMT5-1
sgWDR77-2
DNA content
0 50K 100K 150K 200K 250K
p=0.0136
p=0.0084
p=0.0039
p=0.0014
%
G1
S
G2

Figure S4. Supportive data for negative selection assays and flow cytometry assays. (A) GFP drop-out assays in indicated SCC WT cells expressing Control or PRMT5 CR or WDR77 CR for sgROSA26, sgCDK1, sgPRMT5-1, and sgWDR77-2. Normalized to P0. The *p* values were calculated using One-Way ANOVA. (B) Schematic of PRMT5 CR and WDR77 CR. (C) Confirmation of PRMT5KO and WDR77KO via western blot in SCC scramble cells (control) and SCC Cells overexpressing PRMT5 CR or WDR77 CR. (D) Flow cytometry assays in FaDu cells (upper) and Cal33 cells (bottom) treated with sgNeg, sgPRMT5-1 and sgWDR77-2 (n = 3 biologically independent samples). Data are presented as means ± S.D. The *p* values were calculated using Two-Way ANOVA.

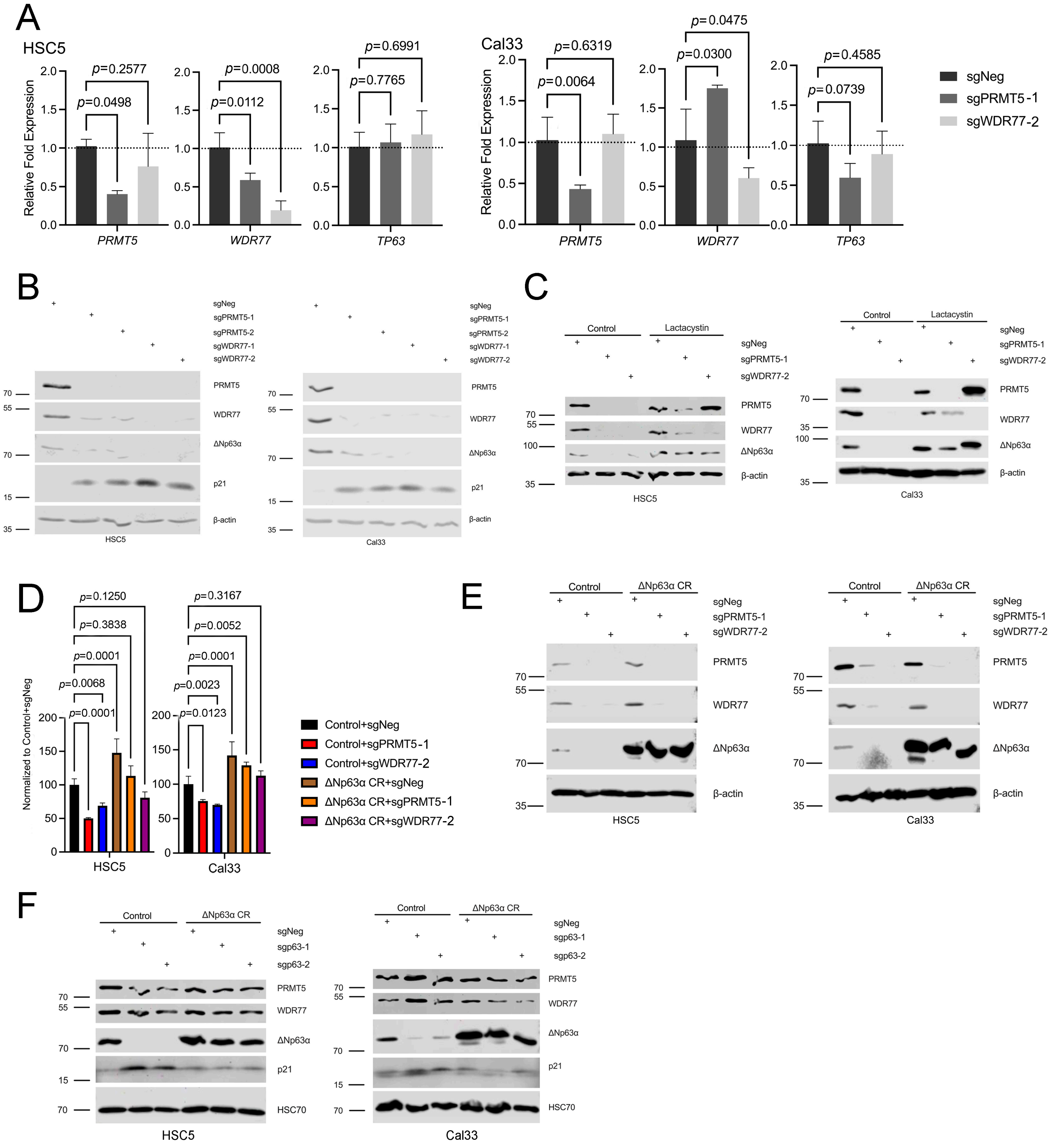

Figure S5. PRMT5 and WDR77 modulates the ΔNp63α-p21 pathway in SCC cell lines. (A) Assessment of *PRMT5*, *WDR77*, and *TP63* transcription levels via qRT-PCR in HSC5 (left) and Cal33 (right) cells treated with sgPRMT5-1 and sgWDR77-2. Normalized to cells treated with sgNeg. The *p* values were calculated using One-Way ANOVA. (B) Assessment of PRMT5, WDR77, ΔNp63α and p21 expression via western blot in HSC5 (left) and Cal33 (right) cells treated with sgNeg (empty vector control), sgPRMT5-1, sgPRMT5-2, sgWDR77-1, and sgWDR77-2. (C) HSC5 (left) or Cal33 (right) cells were infected with sgNeg, sgPRMT5-1, and sgWDR77-2, with or without additional treatment of 1 μmol/L lactacystin for 24 hours. (D) MTT-based proliferation assays in HSC5 (left) and Cal33 (right) scramble cells (control) and corresponding cells overexpressing ΔNp63α CR. Cells were infected with sgNeg, sgPRMT5-1, and sgWDR77-2. Data are presented as means ± S.D., normalized to sgNeg (n = 3 biologically independent samples). The *p* values were calculated using One-Way ANOVA. (E) Confirmation of PRMT5O and WDR77KO via western blot in HSC5 (left) and Cal33 (right) scramble cells (control) and corresponding cells overexpressing ΔNp63α CR. These cells were treated with sgNeg, sgPRMT5-1, and sgWDR77-2. (F) Assessment of PRMT5, WDR77, ΔNp63α and p21 expression via western blot in HSC5 (left) and Cal33 (right) scramble cells (control) and corresponding cells overexpressing ΔNp63α CR. These cells were treated with sgNeg (empty vector control), sgp63-1 and sgp63-2.

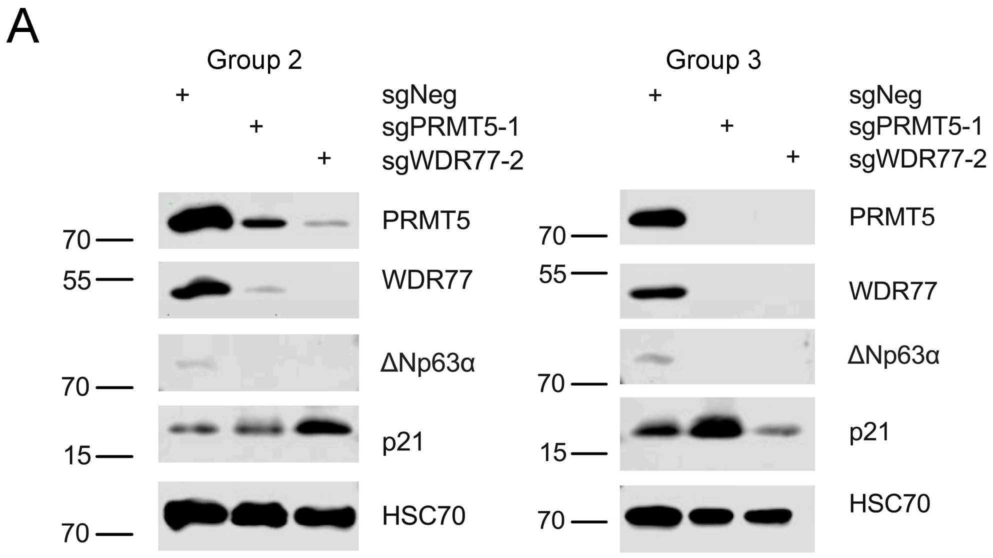

Figure S6. PRMT5 and WDR77 modulates the ΔNp63α-p21 pathway *in vivo*. (A) Assessment of PRMT5, WDR77, ΔNp63α and p21 expression in the tumor samples from mice.

Figure S7. Original western blot pictures.

Related to Figure 4B and Supplementary Figure S5B

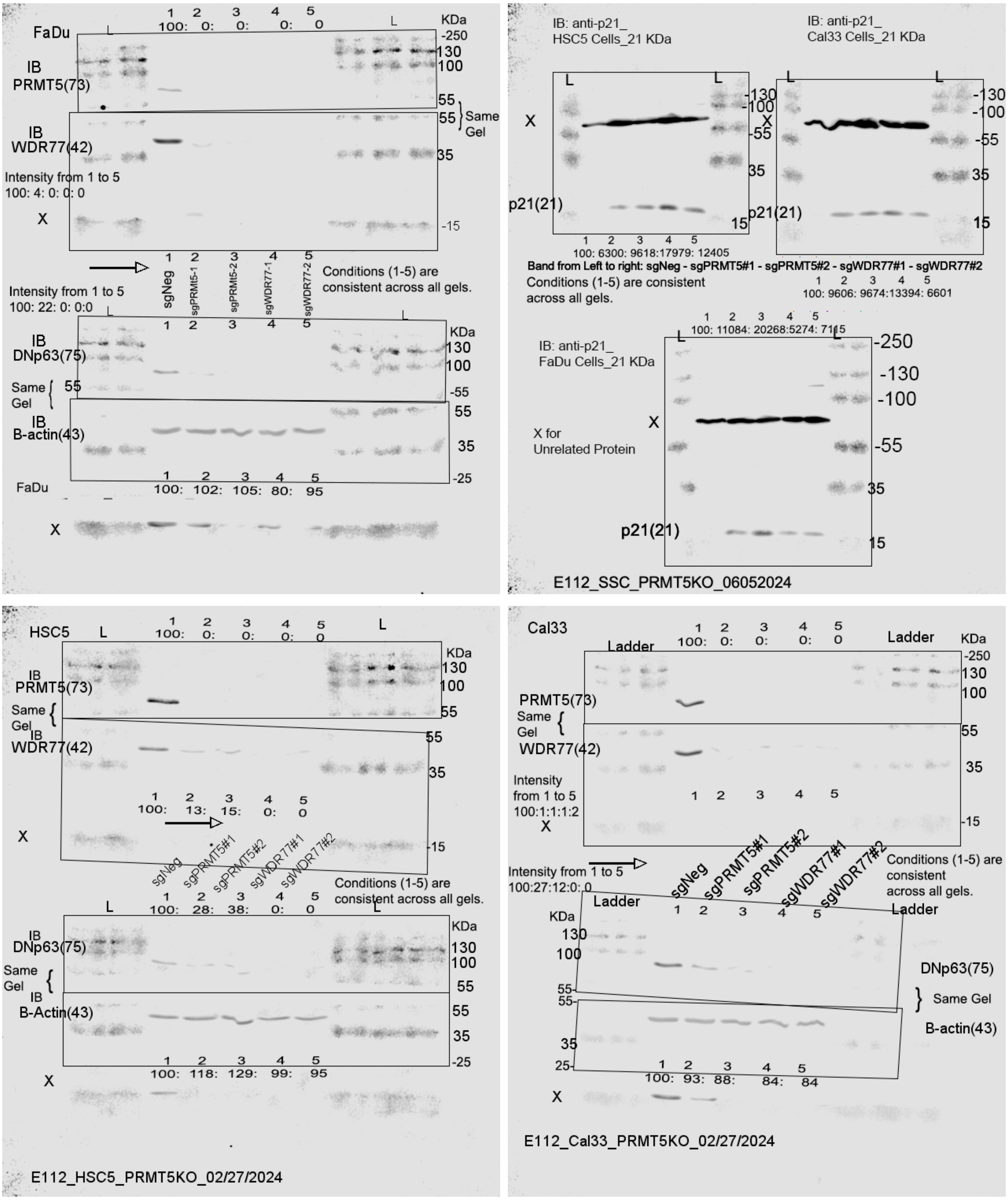

Related to Figure 4C and Supplementary Figure S5C

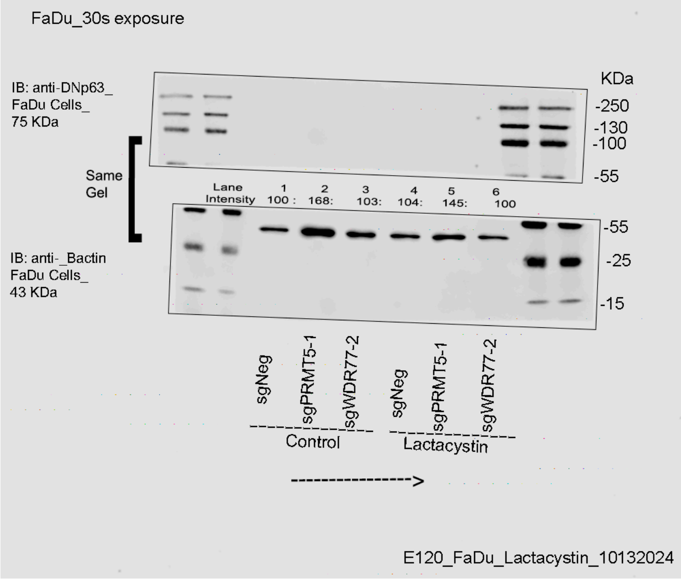
FaDu_30s exposure
IB: anti-DNp63_
FaDu Cells_
75 KDa
Same
Gel
IB: anti-_Bactin
FaDu Cells_
43 KDa
KDa
-250
-130
-100
-55
Lane
Intensity
1
100 :
2
168:
3
103:
4
104:
5
145:
6
100
-55
-25
-15
sgNeg
sgPRMT5-1
sgWDR77-2
sgNeg
sgPRMT5-1
sgWDR77-2
Control
Lactacystin
---------------->
E120_FaDu_Lactacystin_10132024

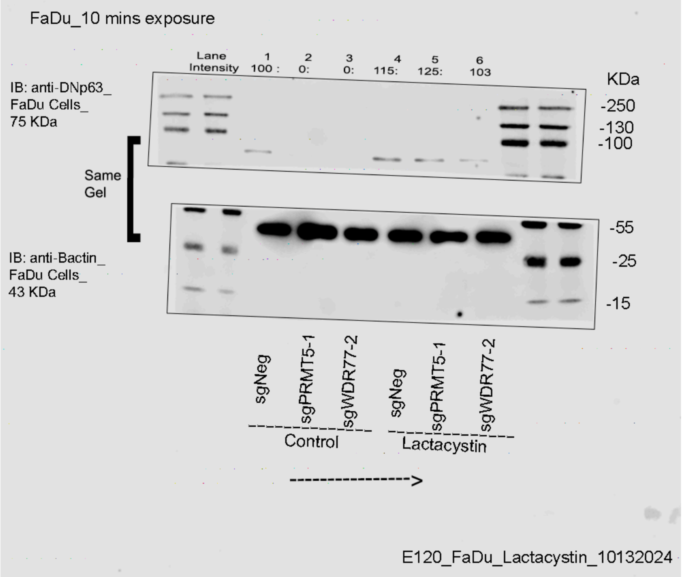
FaDu_10 mins exposure
Lane
Intensity
1
100 :
2
0:
3
0:
4
115:
5
125:
6
103
KDa
IB: anti-DNp63_
FaDu Cells_
75 KDa
-250
-130
-100
Same
Gel
IB: anti-Bactin_
FaDu Cells_
43 KDa
-55
-25
-15
sgNeg
sgPRMT5-1
sgWDR77-2
sgNeg
sgPRMT5-1
sgWDR77-2
Control
Lactacystin
---------------->
E120_FaDu_Lactacystin_10132024

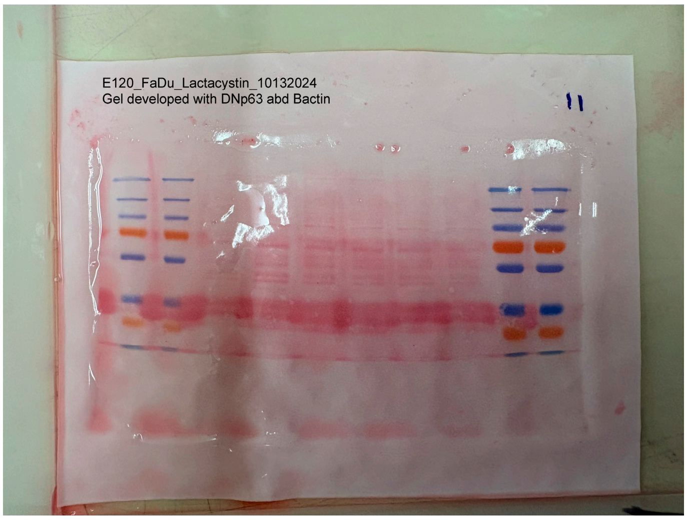
E120_FaDu_Lactacystin_10132024
Gel developed with DNp63 abd Bactin
11

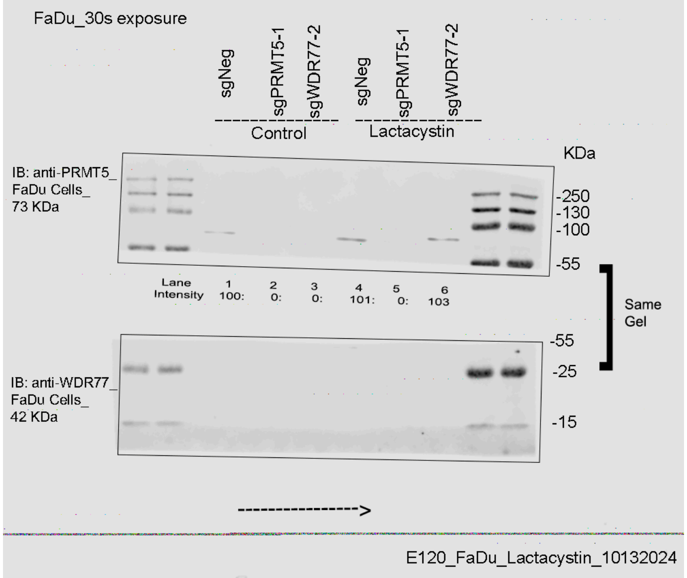
FaDu_30s exposure
sgNeg
sgPRMT5-1
sgWDR77-2
sgNeg
sgPRMT5-1
sgWDR77-2
Control
Lactacystin
KDa
IB: anti-PRMT5_
FaDu Cells_
73 KDa
-250
-130
-100
-55
Lane
Intensity
1
100:
2
0:
3
0:
4
101:
5
0:
6
103
Same
Gel
-55
-25
-15
IB: anti-WDR77_
FaDu Cells_
42 KDa
---------------->
E120_FaDu_Lactacystin_10132024

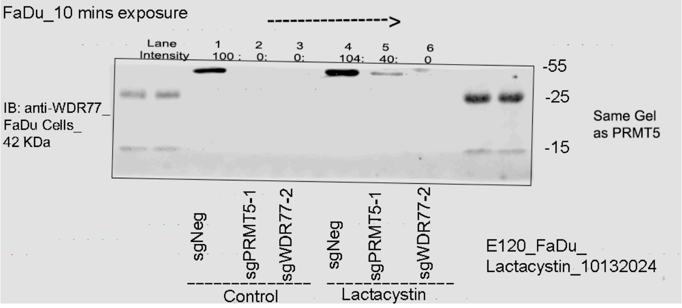
FaDu_10 mins exposure
---------------->
Lane
Intensity
1
100 :
2
0:
3
0:
4
104:
5
40:
6
0
-55
-25
-15
IB: anti-WDR77_
FaDu Cells_
42 KDa
Same Gel
as PRMT5
sgNeg
sgPRMT5-1
sgWDR77-2
sgNeg
sgPRMT5-1
sgWDR77-2
Control
Lactacystin
E120_FaDu_
Lactacystin_10132024

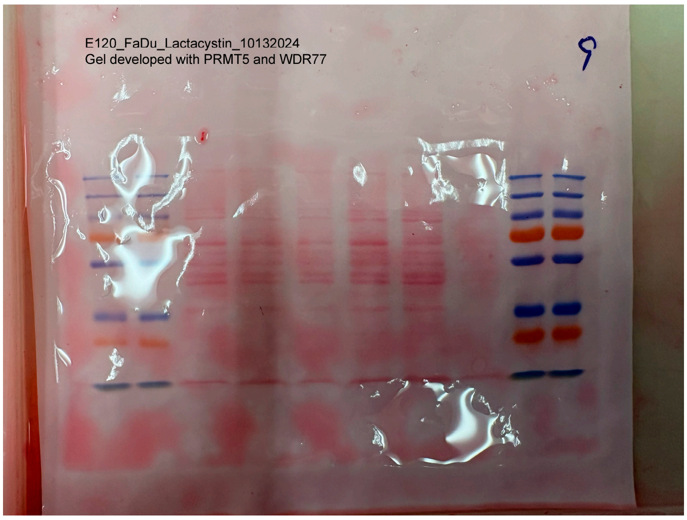
E120_FaDu_Lactacystin_10132024
Gel developed with PRMT5 and WDR77
9

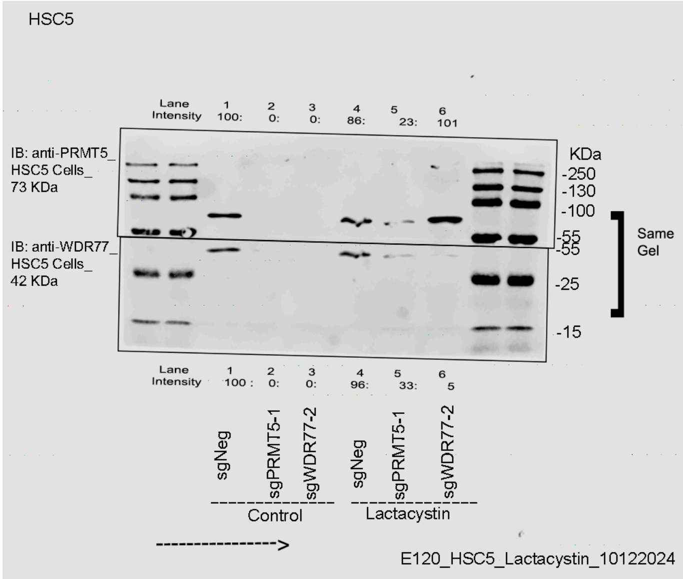
HSC5
Lane
Intensity
1 100:
2 0:
3 0:
4 86:
5 23:
6 101
IB: anti-PRMT5_
HSC5 Cells_
73 KDa
IB: anti-WDR77_
HSC5 Cells_
42 KDa
KDa
-250
-130
-100
-55
-55
-25
-15
Same
Gel
Lane
Intensity
1 100 :
2 0:
3 0:
4 96:
5 33:
6 5
sgNeg
sgPRMT5-1
sgWDR77-2
sgNeg
sgPRMT5-1
sgWDR77-2
Control
Lactacystin
---------------->
E120_HSC5_Lactacystin_10122024

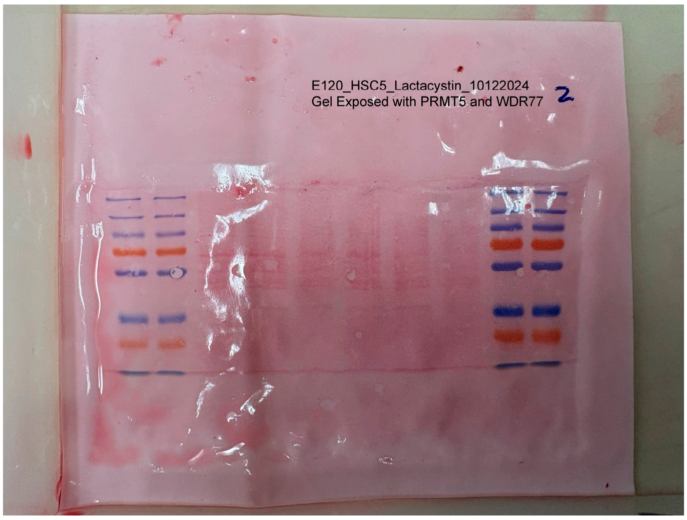
E120_HSC5_Lactacystin_10122024
Gel Exposed with PRMT5 and WDR77
2

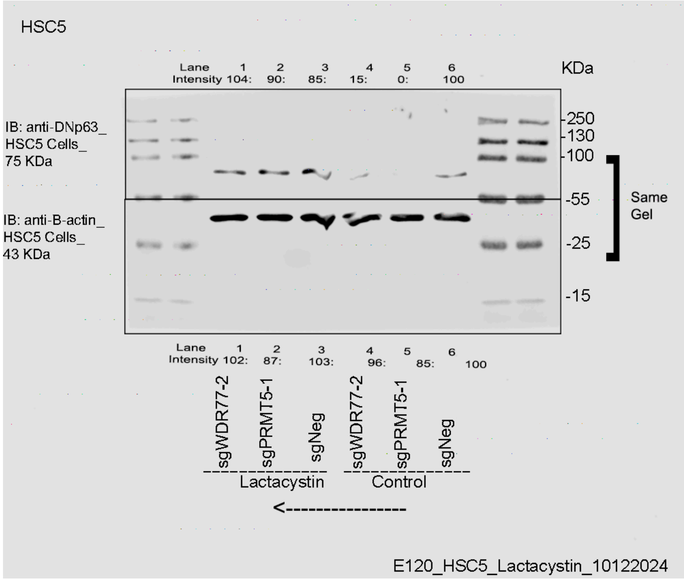
HSC5
Lane
Intensity 104:
1
2 90:
3 85:
4 15:
5 0:
6 100
KDa
IB: anti-DNp63_
HSC5 Cells_
75 KDa
IB: anti-B-actin_
HSC5 Cells_
43 KDa
-250
-130
-100
-55
-25
-15
Same
Gel
Lane
Intensity 102:
1
2 87:
3 103:
4 96:
5 85:
6 100
sgWDR77-2
sgPRMT5-1
sgNeg
sgWDR77-2
sgPRMT5-1
sgNeg
Lactacystin
Control
<----------------
E120_HSC5_Lactacystin_10122024

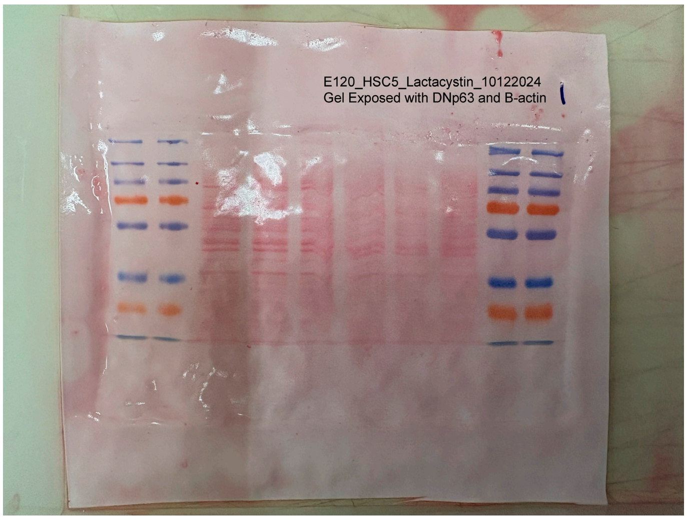
E120_HSC5_Lactacystin_10122024
Gel Exposed with DNp63 and B-actin
1

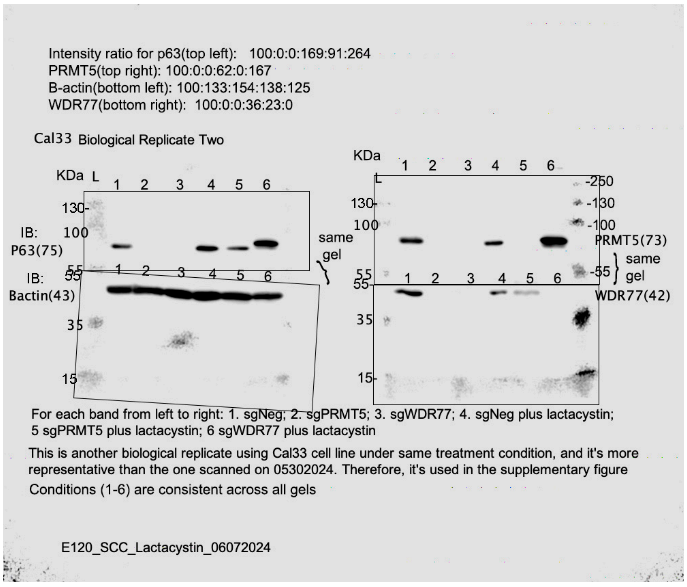

Related to Figure 4E and Supplementary Figure S5E

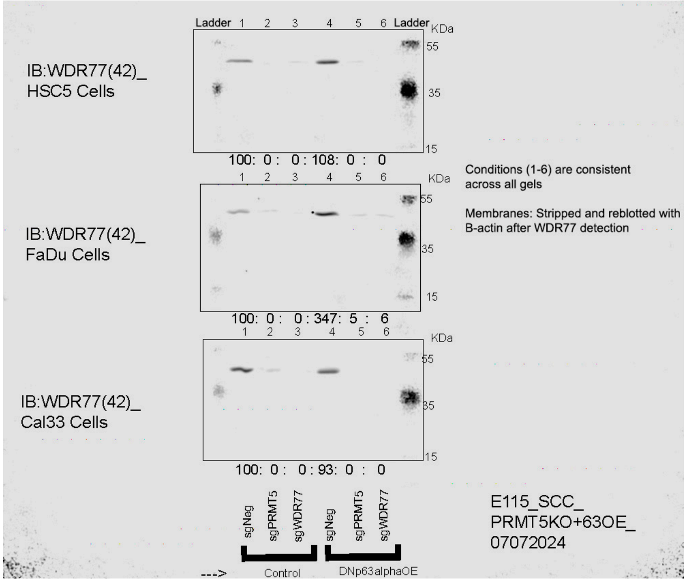

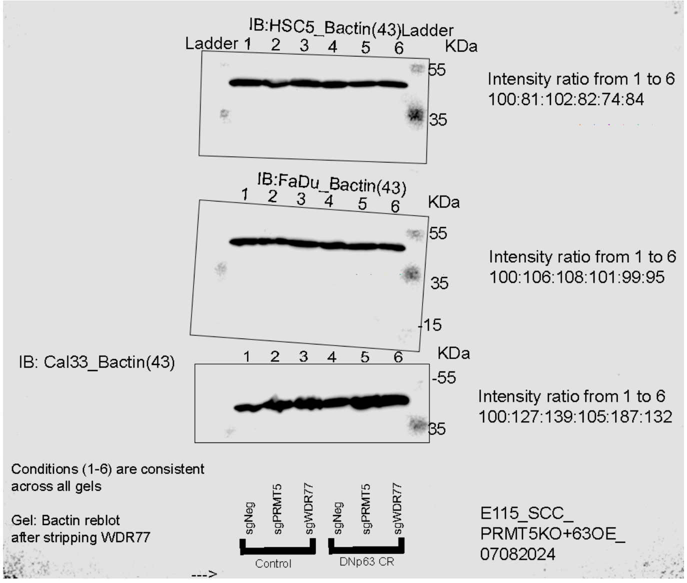

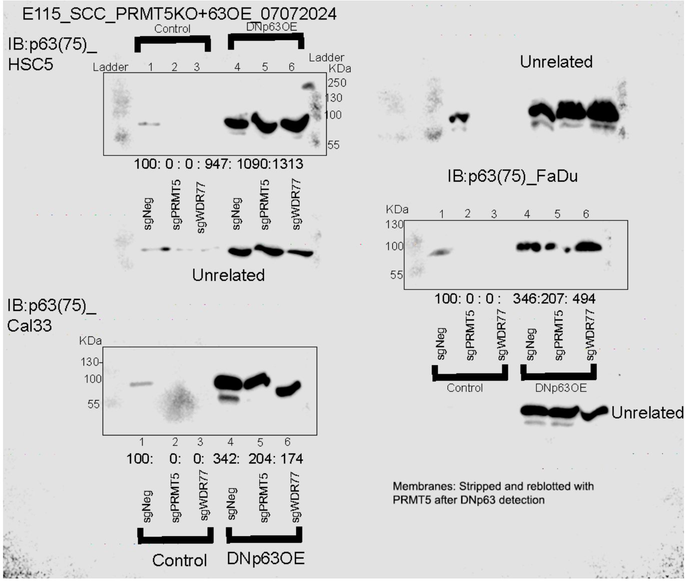

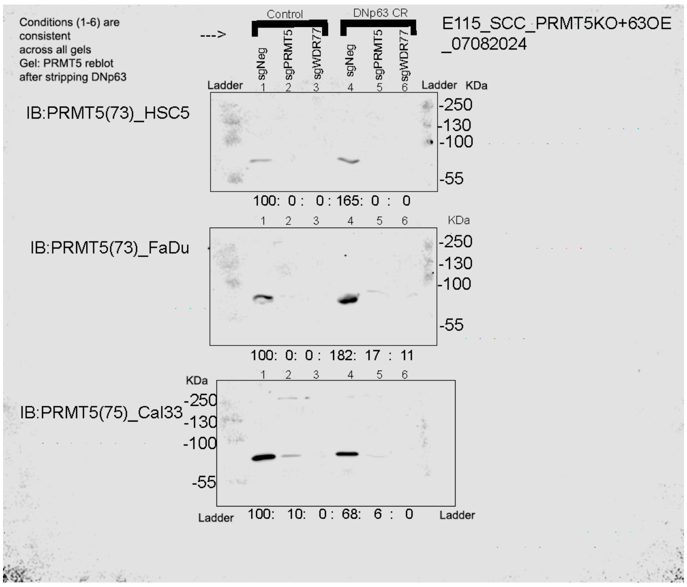

Related to Figure 4F and Supplementary Figure S5F

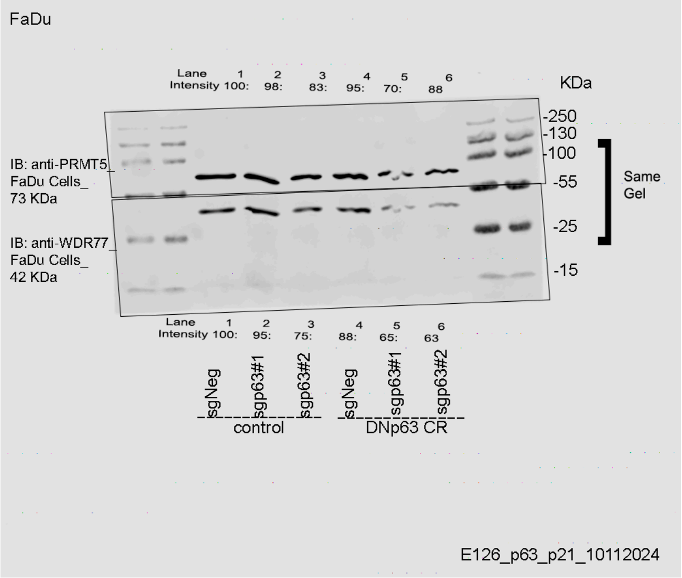

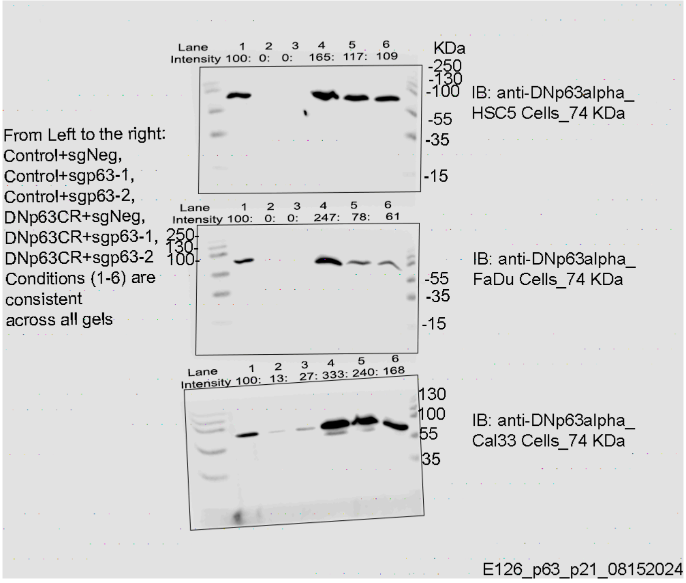

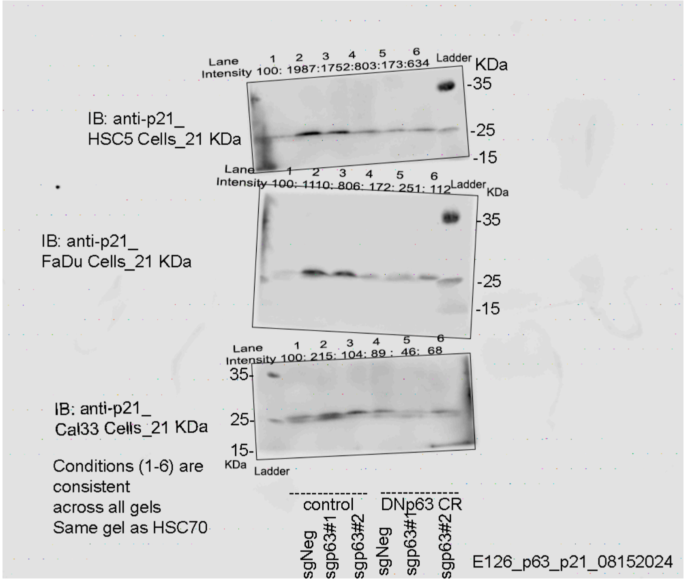

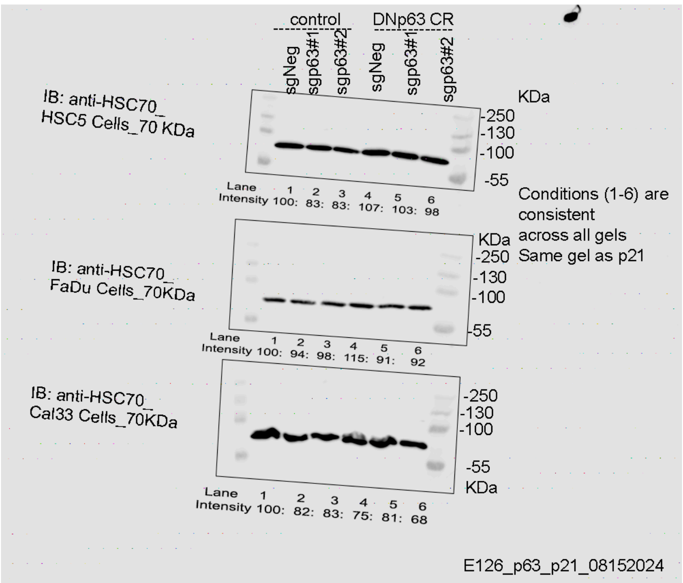

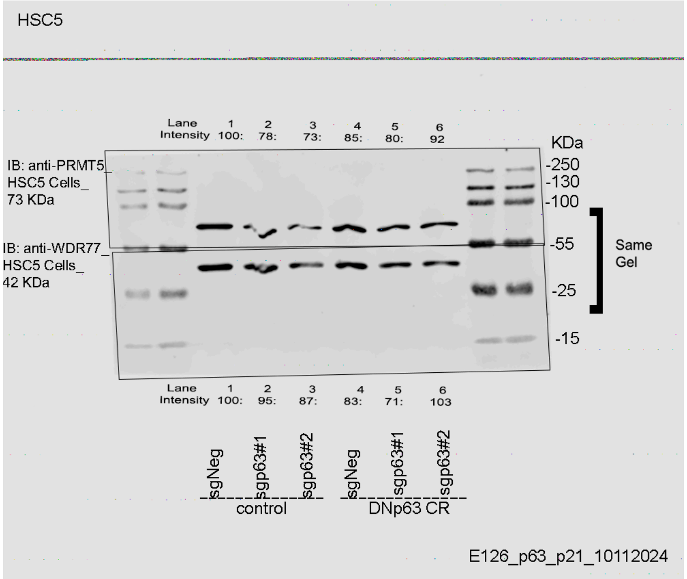

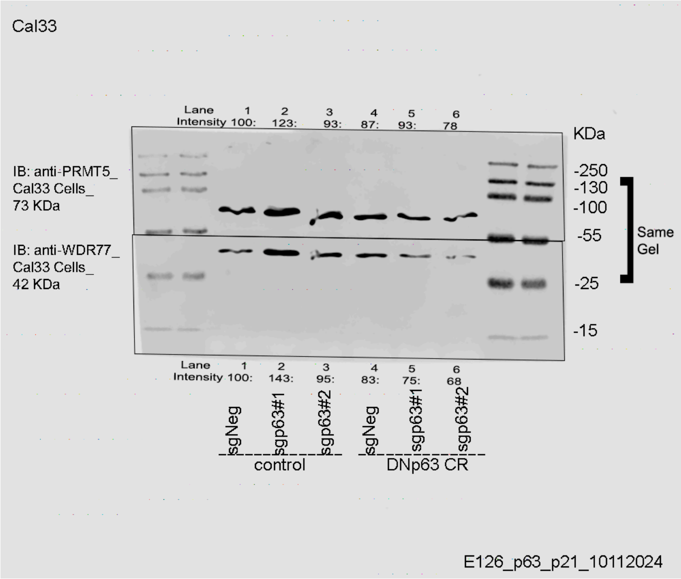

Related to Figure 5D and Supplementary Figure S6

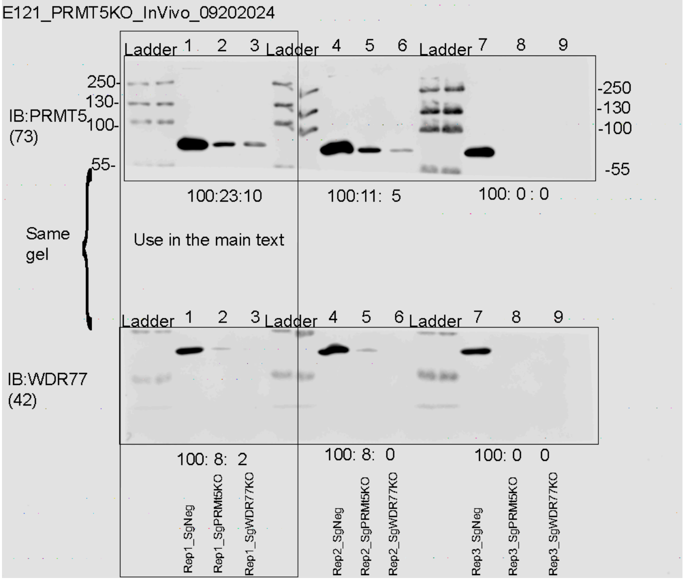

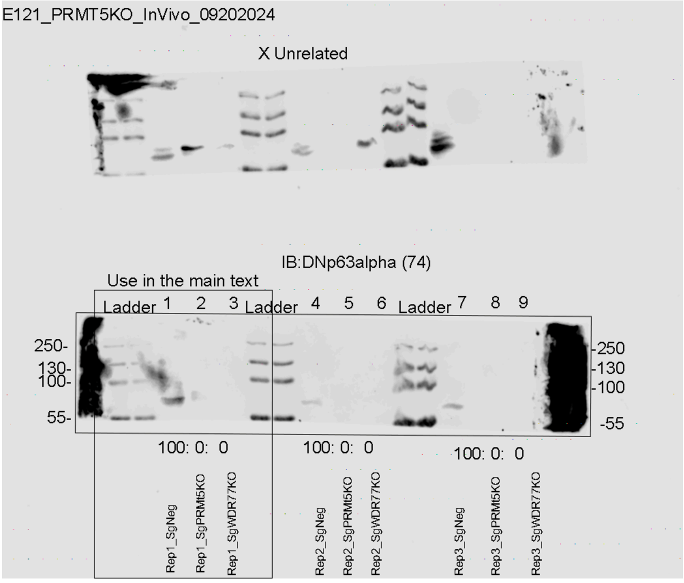

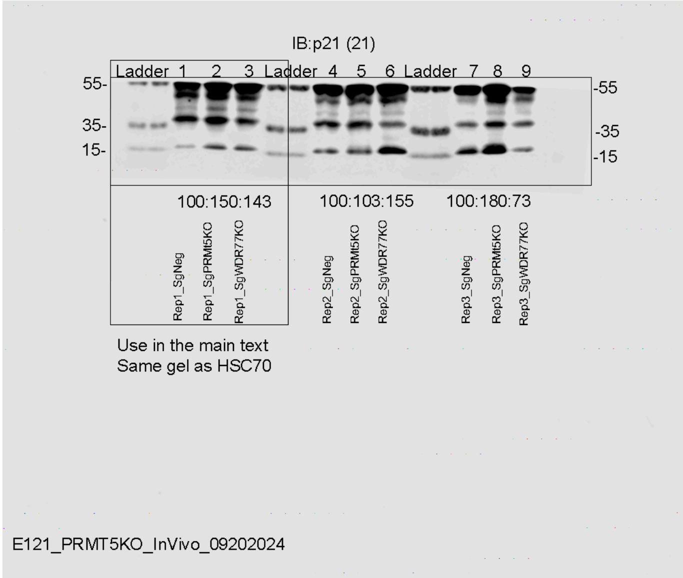

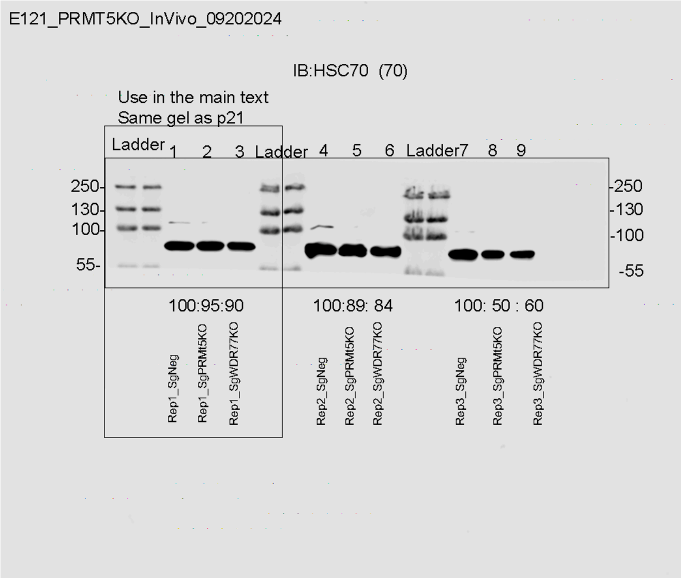

Related to Supplementary Figure S4C

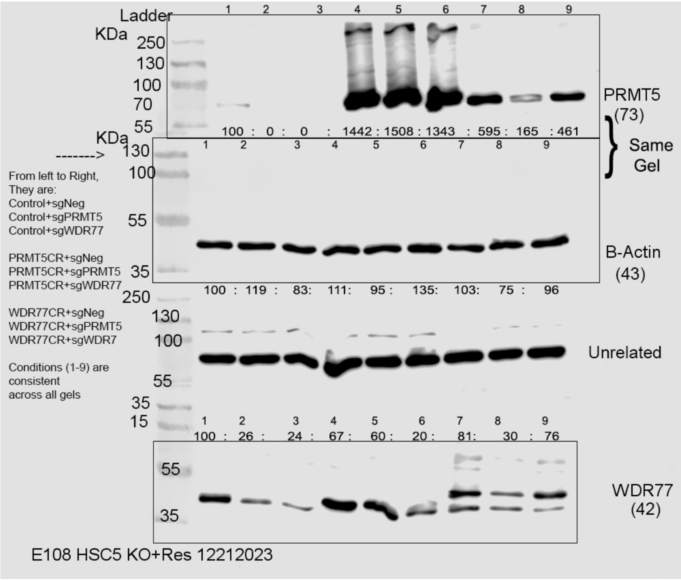

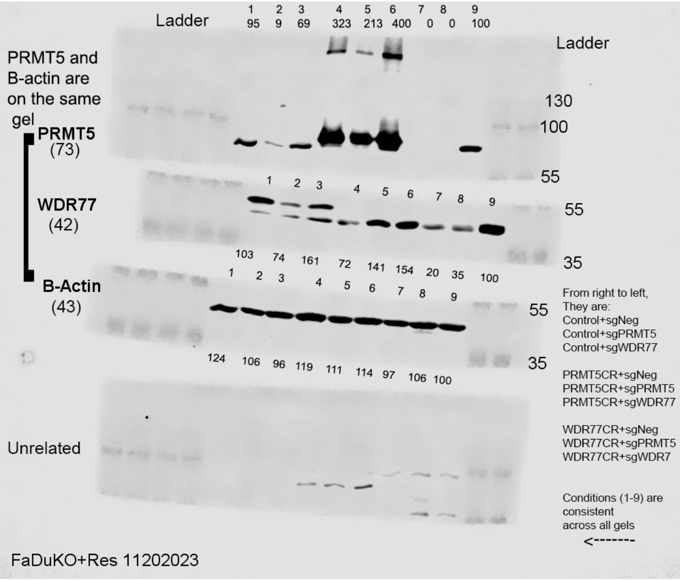

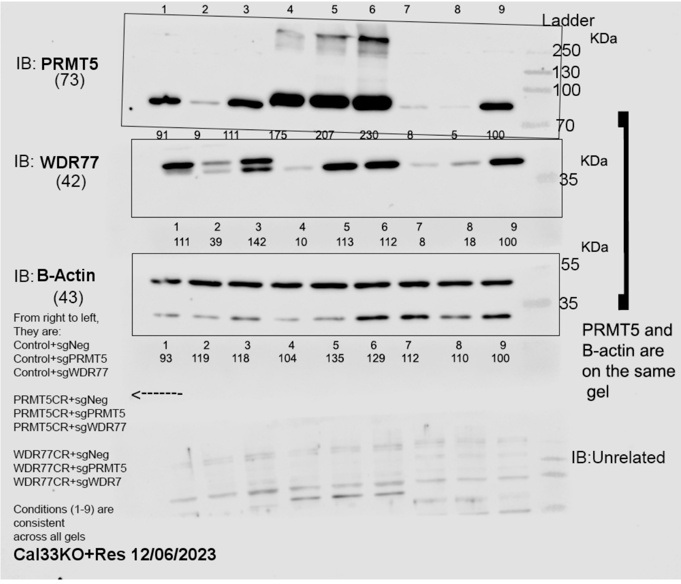

Figure S8. Original images of tumors from mice

sgNeg (4 mice)

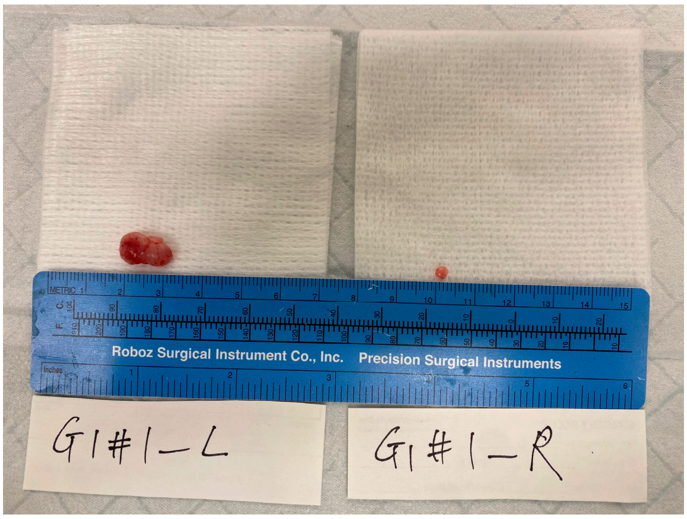

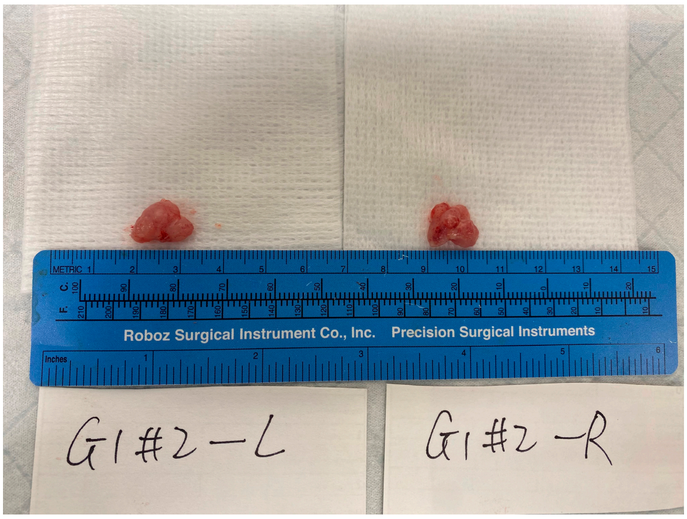

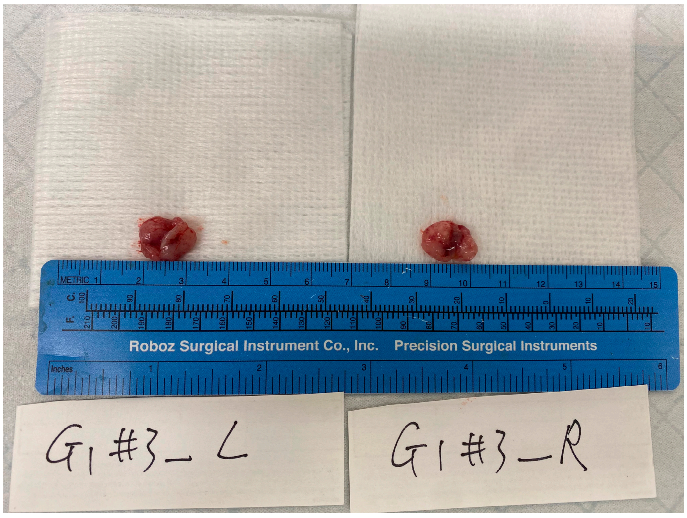

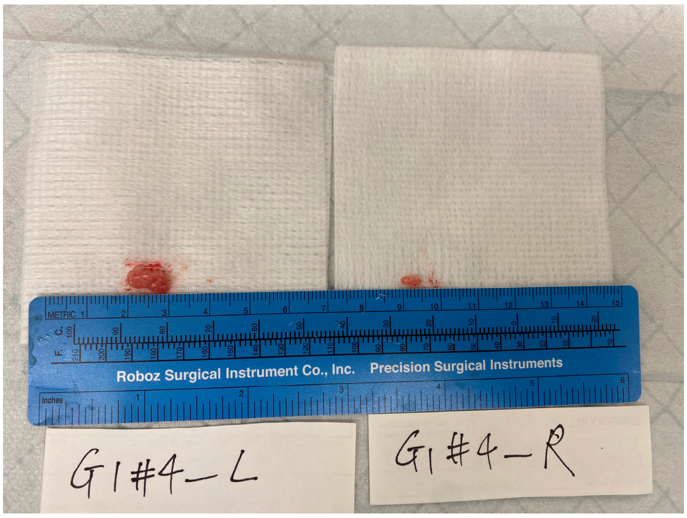

sgPRMT5-1 (4 mice)

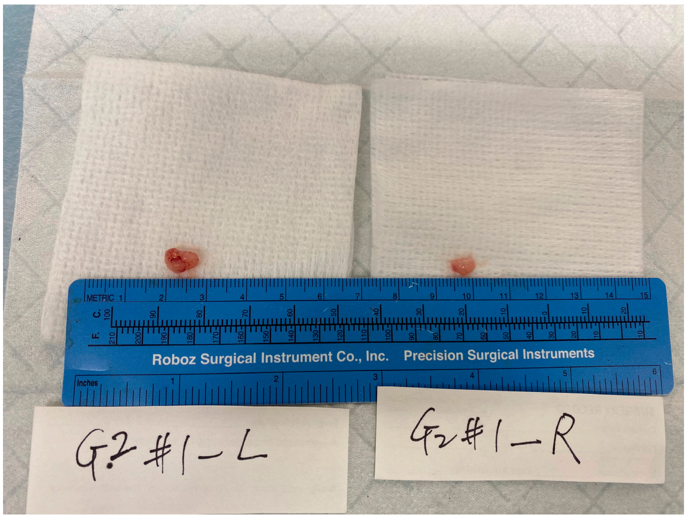

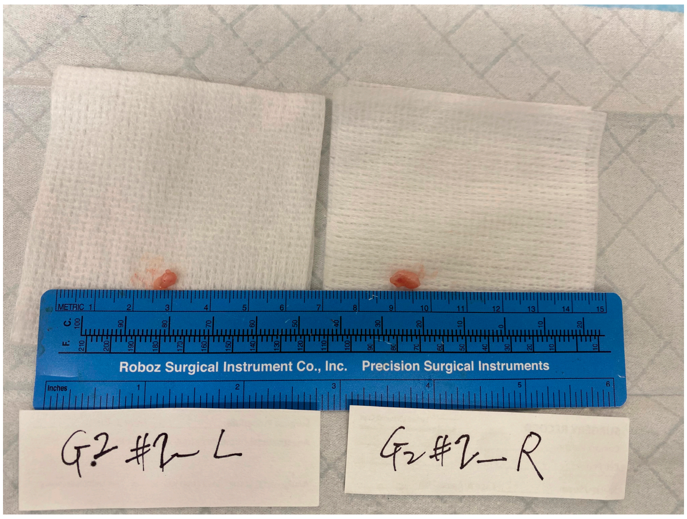

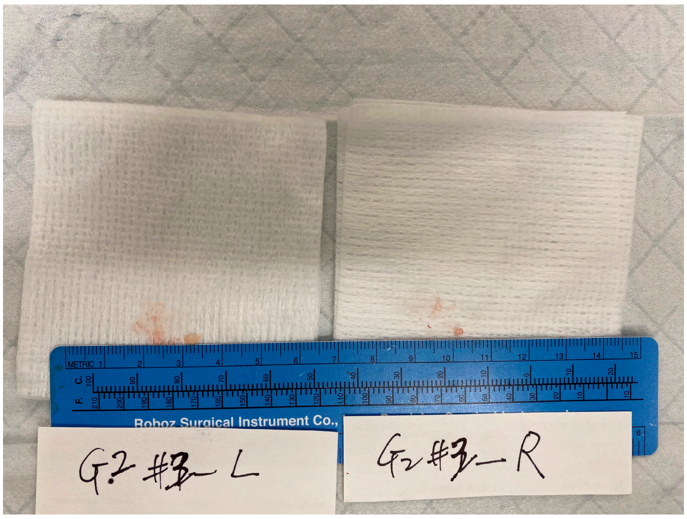

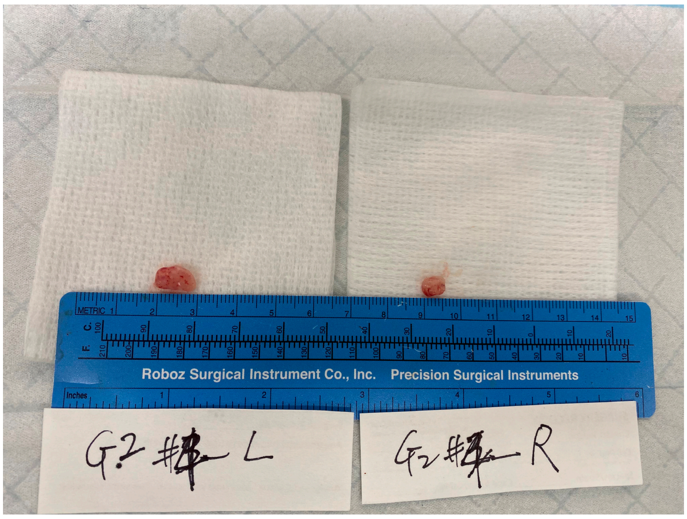

sgWDR77-2 (4 mice, missing picture for mouse number 2)

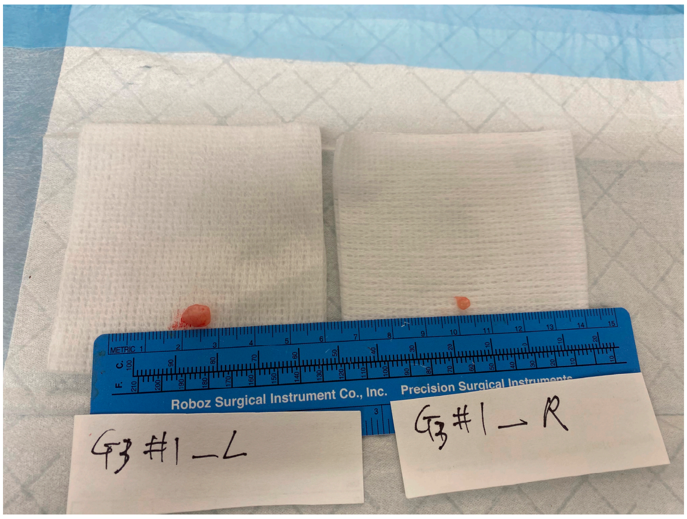

ND

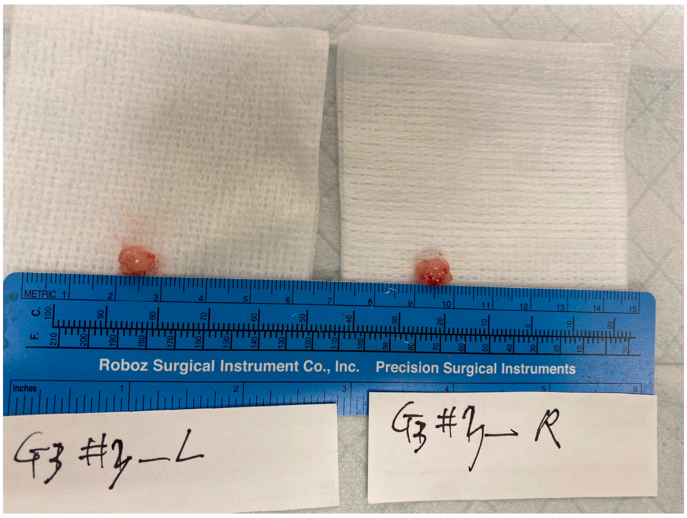

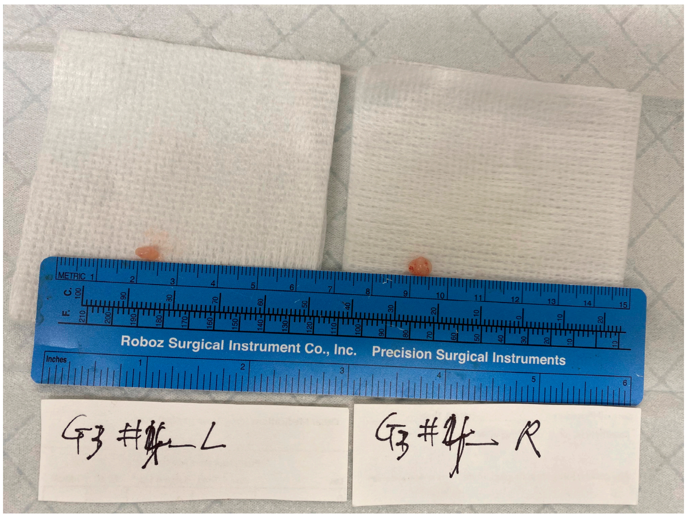

Supplement: Supplementary file 1 [file cancers-16-03789-s001.zip › cancers-3243107-supplementary_Revised HengL.pdf]
